# CREB regulates Foxp3^+^ST-2^+^ T_REGS_ with enhanced IL-10 production

**DOI:** 10.3389/fimmu.2025.1601008

**Published:** 2025-07-24

**Authors:** Sudheendra Hebbar Subramanyam, Judit Turyne Hriczko, Saskia Schulz, Thomas Look, Tannaz Goodarzi, Tim Clarner, Miriam Scheld, Markus Kipp, Eva Verjans, Svenja Böll, Christopher Neullens, Ivan Costa, Zhijian Li, Lin Gan, Bernd Denecke, Angela Schippers, Stefan Floess, Jochen Huehn, Edgar Schmitt, Tobias Bopp, Hermann Wasmuth, Ron Winograd, Rudi Beyaert, Bart Lambrecht, Martin Zenke, Norbert Wagner, Kim Ohl, Klaus Tenbrock

**Affiliations:** ^1^ Department of Pediatrics, Rheinisch-Westfälische Technische Hochschule Aachen University Hospital, Aachen, Germany; ^2^ Helmholtz-Institute for Biomedical Engineering, Rheinisch-Westfälische Technische Hochschule Aachen University, Aachen, Germany; ^3^ Institute for Biomedical Engineering, Department of Cell Biology, Rheinisch-Westfälische Technische Hochschule Aachen University Hospital, Aachen, Germany; ^4^ Institute of Neuroanatomy and Jülich Aachen Research Alliance (JARA)-BRAIN, Faculty of Medicine, Rheinisch-Westfälische Technische Hochschule Aachen University, Aachen, Germany; ^5^ Institute of Anatomy, Medical University of Rostock, Rostock, Germany; ^6^ Instutute for Computational Genomics, Interdisciplinary Center for Clinical Research Aachen (IZKF Aachen), Rheinisch-Westfälische Technische Hochschule Aachen University Hospital, Aachen, Germany; ^7^ Genomics Facility, Interdisciplinary Center for Clinical Research Aachen (IZKF Aachen), Rheinisch-Westfälische Technische Hochschule Aachen University Hospital, Aachen, Germany; ^8^ Department of Experimental Immunology, Helmholtz Centre for Infection Research, Braunschweig, Germany; ^9^ Institute for Immunology, University Medical Center, Johannes Gutenberg University Mainz, Mainz, Germany; ^10^ Department of Medicine, Luiusenhospital Aachen, Aachen, Germany; ^11^ Vlaams Instituut voor Biotechnologie (VIB) Center for Inflammation Research, Ghent, Belgium; ^12^ Department of Biomedical Molecular Biology, Ghent University, Ghent, Belgium; ^13^ Department of Respiratory Medicine, Ghent University Hospital, Ghent, Belgium; ^14^ Department of Medicine IV, Hematology, Oncology, and Stem Cell Transplantation, Rheinisch-Westfälische Technische Hochschule Aachen University Hospital, Aachen, Germany; ^15^ Center for Integrated Oncology Aachen Bonn Cologne Duesseldorf (CIO ABCD), Aachen, Germany; ^16^ Division of Pediatric Rheumatology, Department of Pediatrics, Inselspital, Bern University Hospital, University of Bern, Bern, Switzerland

**Keywords:** IL-33, ST2, Foxp3, IL-10, CREB, CREM

## Abstract

**Introduction:**

Regulatory T-cells (T_regs_) are characterized by the expression of Foxp3, a master regulator involved in the development and function of T_regs_. Foxp3 expression is dependent on activity of the Treg specific demethylated site (TSDR), which contains a CREB binding site. We aimed to find out how Foxp3 specific CREB deletion affects Treg expression and function.

**Methods:**

T_regs_ from *Foxp3^cre^CREB^fl/fl^* mice and wild type (*CREB^fl/fl^*) mice were analyzed by flow cytometry. Cytokine analysis was performed by flow cytometry, ELISA and RT-qPCR. Gene expression analysis was performed using Affymetrix HTA2 assays, ATAC-sequencing, and Methylation-assays. For functional relevance, a CD4 T cell mediated transfer colitis was performed.

**Results and discussion:**

*Foxp3^cre^CREB^fl/fl^* mice showed increased frequencies of T_regs_ (CD25+/Foxp3+) in thymus, spleen and peripheral lymph nodes and in nonlymphoid organs including lung and colon, but decreased Foxp3 expression at the single cell level. Despite decreased Foxp3 expression, enhanced expression of the IL- 33 receptor (ST-2), IL-10, IL-13, and CREM was observed. CREB deficient T_regs_ were highly suppressive *in vitro* and prevented disease activity in a CD4 T cell mediated transfer colitis in an IL-10 dependent way. Mechanistically CREB fulfils dual roles in T_regs_: (1) it promotes Foxp3 expression under Steady state conditions and (2) in cooperation with CREM, CREB restricts chromatin accessibility at the ST2 locus, thereby modulating IL-33 driven immune responses. This dual regulation balances FoxP3-dependent Treg stability with IL-10 mediated suppression of inflammation.

## Graphical Abstract

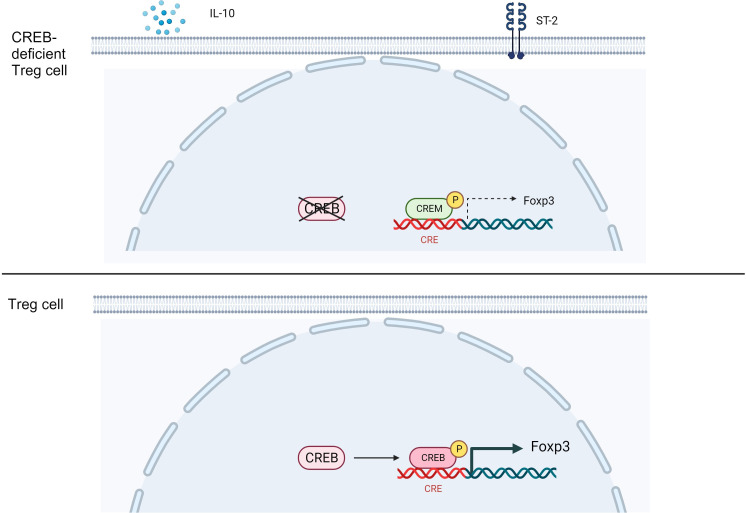

## 1 Introduction

Regulatory T cells (Tregs) marked by Foxp3 expression are important for immune balance. In the absence of Foxp3, mice (scurfy disease) (1) and humans (IPEX, polyglandular autoimmunopathy) develop severe autoimmune disease (2). Foxp3 Tregs arise from two sources; thymus-derived Tregs (t-Tregs) develop in the thymus, while inducible Tregs (iTregs) are generated in the periphery from conventional CD4+ T cells upon TGF-β stimulation (3). Both tT_reg_ and iT_reg_ generation largely depend on the presence of IL-2, which is not only required for homeostatic maintenance but also for the thymic development of T_regs_ (4–7).

ST-2, also known as IL1RL1, belongs to the Toll-like receptor/IL-1 receptor superfamily. ST-2, through its ligand IL-33, plays a pivotal role in MyD88/NFκB signaling. The interaction of ST-2 with its ligand IL-33 induces Foxp3 and GATA3 expression in T_regs_ at mucosal sites (8). ST-2 mainly exists in two isoforms: soluble ST-2 (sST-2) and membrane-bound ST-2. sST-2 functions as a decoy receptor of IL-33 and inhibits NFκB signaling, while membrane-bound ST-2 promotes NFκB signaling (9). Increased ST-2 levels have been observed in diseases such as inflammatory bowel disease (IBD), asthma, colon cancers, and graft versus host disease (10). In many non-lymphoid tissues, T_regs_ show enriched expression of ST-2 and most of these are iT_regs_. The regulation of ST-2^+^T_regs_ is dependent on the transcription factor basic leucine zipper transcription factor ATF-like (BATF) (11, 12), while other factors have not yet been defined. BATF belongs to the cAMP-responsive element binding protein/activating transcription factor (CREB/ATF) family of transcription factors, can dimerize with Jun, and has been shown to compete with CREB for binding to CRE/AP1 sites.

Our understanding of the molecular mechanism of suppression is still limited. IL-10 plays an important role in suppressing CD4^+^ effector T-cell function. Additionally, IL-10 prevents activation of antigen-presenting cells (APCs), such as dendritic cells and macrophages, through the downregulation of costimulatory molecules and production of inflammatory cytokines within these cells (13). Selective ablation of IL-10 in Foxp3^+^ T_regs_ revealed that IL-10 production by T_regs_ is essential for keeping the immune response in check at environmental interfaces such as the colon and lungs (14). IL-10 also acts in an autocrine manner by maintaining FoxP3 expression and the suppressive capacity of T_regs_ (15, 16). Interestingly, IL-10 is responsive to cAMP stimulation, at least in THP1 cells and macrophages, and the IL-10 promoter contains four putative cAMP response element sites (CRE, TGACGTCA), which are conserved between humans and mice (17). Following TLR activation, pCREB (phosphorylated CREB) is recruited to the IL-10 promoter and enhances IL-10 transcription in macrophages (18).

Previous data from Wang et al. (19) suggested that CREB negatively regulated the survival of iT_regs_ and is particularly important in the generation of CD4+Th17 cells. Using CD4^CRE^ CREB^fl/fl^ mice, they showed that these cells prevented colitis in a Rag2^-/-^ model; however, their model had the disadvantage that the CD4 cells of these mice expressed lower amounts of IL-2, which could have hampered iT_reg_ induction. Our analysis now expands upon their findings and determines an important role for CREB in the generation of ST2+T_regs_. Using Foxp3^CRE^CREB^fl/fl^ mice, we herein confirm the colitis phenotype but can show that i) CREB deficiency in T_regs_ induces expression of ST2+T_regs_, ii) the phenotype that prevents colitis in Rag2^-/-^ mice is primarily dependent on abundance of IL-10, and iii) this phenotype can be reversed by knockout of CREM, which lowers the expression of ST2.

## 2 Materials and methods

### 2.1 Mouse strains

Experiments were performed with age-matched *CREB^fl/fl^, VAV^cre^CREB^fl/fl^*, and *Foxp3^cre^CREB^fl/fl^* mice (all C57BL/6). *Foxp3^cre^CREB^fl/fl^* mice were generated by crossing CREB-flox mice (20) with C57BL/6 Foxp3-IRES Cre mice (provided by T. Bopp, University of Mainz, Germany). *Foxp3^cre-^CREB^fl/fl^* mice were used as controls (denoted as *CREB^fl/fl^*). Foxp3^Cre^ROSA^RFP^ mice were provided by T. Bopp University of Mainz and crossed with our *CREB^fl/fl^* mice. Rag2^-/-^ (C57BL/6) mice were provided by A. Schippers (Uniklinik RWTH Aachen, Germany). All the mice were bred in our animal facility and kept under standardized conditions.

### 2.2 Transfer colitis

To induce transfer colitis, Rag2^-/-^ mice were adoptively transferred 2 x 10^6^ CD4^+^ CD25^-^ T cells. Animals were sacrificed as soon as a significant loss of weight was measurable. The spleen and mesenteric lymph nodes (mLNs) were harvested for further analysis. One part of the colon was fixed in formalin for histological scoring and the other part was fixed in RNAlater (Qiagen, Germany) for subsequent mRNA analysis. For soluble ST2 (sST2) treatment, the mice received 100 µg mouse sST2 (produced by the VIB Protein Core facility, Belgium, and provided by R. Beyaert, VIB-UGent Center for Inflammation Research, Belgium), three times/week by intraperitoneal means (i.p). The control group received PBS i.p. For IL-10R treatment, the mice received 500 µg anti-IL-10R antibody (1B1mAB) (kindly provided by Prof. Edgar Schmitt, University of Mainz, Germany) or IgG control AK (Rat IgG kappa) i.p. once a week during the first 3 weeks.

### 2.3 Histological scoring

Initially, 4 µm paraffin sections from the fixed colon were cut serially, mounted onto glass slides, and deparaffinized. The colon sections were stained with hematoxylin and eosin by the Core Facility (IZKF) of the RWTH Aachen University. Blinded histological scoring was performed using a standard microscope, based on the JLS method as described previously (21, 22). Each colon section was scored for the four general criteria: severity, degree of hyperplasia, degree of ulceration, if present, and percentage of area involved. A subjective range of 1–3 (1 = mild, 2 = moderate, 3 = severe) was used for the first three categories. Severity: Focally small or widely separated multifocal areas of inflammation limited to the lamina propria were graded as mild lesions (1). Multifocal or locally extensive areas of inflammation extending to the submucosa were graded as moderate lesions (2). If the inflammation extended to all layers of the intestinal wall or the entire intestinal epithelium was destroyed, lesions were graded as severe (3). Hyperplasia: Mild hyperplasia consisted of morphologically normal lining epithelium that was at least twice as thick (length of crypts) as adjacent or control mucosa. Moderate hyperplasia was characterized by the lining epithelium being two or three times the normal thickness, cells were hyperchromatic, numbers of goblet cells were decreased, and scattered individual crypts developed an arborizing pattern. Severe hyperplastic regions exhibited a markedly thickened epithelium (four or more times normal thickness), marked hyperchromasia of cells, few to no goblet cells, a high mitotic index of cells within the crypts, and numerous crypts with an arborizing pattern. Ulceration was graded as: 0 = no ulcer, 1 = 1–2 ulcers (involving up to a total of 20 crypts), 2 = 1–4 ulcers (involving a total of 20–40 crypts), and 3 = any ulcers exceeding the former in size. A 10% scale was used to estimate the area involved in the inflammatory process. 0 = 0%, 1 = 10%–30%, 2 = 40%–70%, 3 = >70%.

### 2.4 Cell isolation

Single cell suspensions were isolated from spleens and lymph nodes (LNs) [peripheral lymph nodes (pLNs) and mLNs] using cell strainers and erythrocytes were lysed with lysis buffer. To obtain immune cells from the lungs, tissue was excised into small pieces and digested with 0.1% collagenase in DPBS for 1hr. The digested fraction was passed through a 40μm nylon strainer and rinsed with DPBS. The flow through was centrifuged and the cell pellet was lysed using RBC lysis buffer. The cell pellet was used for the flow cytometric analysis. Colons were retrieved from the mice and rinsed with a cannula with PBS+0.5%BSA. Afterwards, the colons were excised into small pieces and placed in a digestive medium containing RPMI (10mL), collagenase V (0.85mg/mL), collagenase D (1.25mg/mL), dispase (1mg/mL) and DNase (30μg/mL) and incubated for 45 minutes at 37°C with gentle shaking. The digested fraction was passed through a 40 μm nylon strainer and the flow-through was collected and centrifuged to obtain a pellet. Percoll gradient centrifugation was performed by using 35% Percoll. The cell pellet obtained was lysed using RBC lysis buffer and was used for further analysis. The livers were retrieved from the mice. The gall bladders were removed and the livers were excised into small pieces and placed in a digestive medium containing RPMI without FCS (3mL), 1.25mg/mL collagenase D, and 30μg/mL DNase. The fraction was incubated for 45 minutes and digestion was stopped by adding PBS+0.5% BSA+2mM EDTA and homogenized using a syringe. The digested fraction was passed through a 100μm nylon strainer and the flow-through was centrifuged. The pellet obtained was used for the Percoll gradient centrifugation using 35% Percoll. The cell pellet obtained after the Percoll gradient centrifugation was lysed using RBC lysis buffer and was used for the flow cytometric analysis.

### 2.5 Suppression Assay

CD4 T-cells (T_effector_ cells) were isolated from the spleens of wild-type mice. The cells were labeled with eFluor 660 proliferation dye from Thermo Fischer according to the manufacturer’s instructions. After the labeling, the cells were stimulated with plate-coated anti-CD3 (10µg/mL, eBioscience, Germany) and soluble anti-CD28 (1µg/mL) (eBioscience) in the presence of CD4+CD25+ cells from either wild-type or *Foxp3^cre^CREB^fl/fl^* mice in different ratios (1:0, 1:0.5, 1:0.75, and 1:1) for 3 days. After 3 days of stimulation, the cells were analyzed using flow cytometric analysis.

### 2.6 T cell differentiation assays

Magnetic Activated Cell Sorting (MACS)-isolated CD4^+^CD25^-^ T cells (2x10^6^ per mL) were incubated with plate-coated anti-CD3 (10µg/mL, eBioscience, Germany) and soluble anti-CD28 (1µg/mL) (eBioscience, Germany). T_H_0 cells were left without exogenous cytokines, and 5 ng/mL TGF-β was added to induce T_reg_ differentiation.

### 2.7 Flow cytometry

For surface staining, single cell suspensions were stained with anti-CD4, anti-CD3, anti-CD8, anti-B220, anti-CD25, CD45, anti-GL-7, anti-ICOS, anti-ST2, anti-Nrp1, and anti-PD-1 (all from eBioscience, Germany). For intracellular staining of CREB, Foxp3, CTLA-4, Helios, GATA3, T-bet, and RORγt, cells were fixed and permeabilized with a FOXP3 staining buffer set (Thermo Fischer, eBioscience, Germany) following the manufacturer’s instructions and stained with respective antibodies (eBioscience, Germany) for 30 min. Intracellular cytokines were stained with anti-IFN-γ-Alexa 647 (eBioscience, Germany) and anti-IL-17-Alexa 488 (BD, USA), and IL-10-APC (eBioscience, Germany) after PMA (30nM) and Ionomycin (1.5µM) (both Sigma-Aldrich, USA) re-stimulation in the presence of GolgiPlug/GolgiStop (BD Biosciences, USA). A daily calibrated FACS-Canto II flow cytometer (Becton Dickinson, MountainView, CA, USA) was used to perform the phenotypic analysis. Lymphocytes were gated by forward (FSC), side scatter (SSC), and CD3, CD4, and B220 expression. A figure exemplifying the gating strategies is provided in the **Supplementary Materials** (**Supplementary Figure 6**). For data analysis FCS-Express 4.0 Research Edition (DeNovo software Glendale, CA, USA) and FlowJo version 10 were used.

### 2.8 Luciferase assay

RLM cells carrying a stable pGL3-TSDR-FoxPro luciferase plasmid were kindly provided by Jochen Huehn (HZI Braunschweig, Germany). Cells were transfected with the pcDNA-CREB plasmid or pcDNA. Cells were left to rest overnight before luciferase activity was measured by using the Dual-Glo Luciferase Assay System (Promega, USA).

### 2.9 RNA isolation and real-time PCR

Total RNA from isolated T cells and colon tissue was isolated using the RNeasy Mini Kit (Qiagen, Germany). cDNA was then generated from 200 ng total RNA using the RevertAid H Minus First Strand cDNA Synthesis Kit (Thermo Fisher Scientific, USA) according to the manufacturer´s instructions. RT-PCR was performed using the SYBR Green PCR kit (Eurogentec, Germany) and data were acquired with the ABI Prism 7300 RT-PCR system (Applied Biosystems/Life Technologies, Germany). Each measurement was set up in duplicate. After normalization to the endogenous reference control gene ß-actin for mice, the relative expression was calculated.

### 2.10 RNA extraction and microarray for gene expression analysis

CD4^+^CD25^+^Nrp1^+^ T_regs_ were sorted by FACS. Genome-wide transcriptome analyses of Foxp*^cre^CREB^fl/fl^* and *CREB^fl/fl^* T_regs_ were performed in independent triplicate using Gene Chip^®^ Mouse Gene 2.0 arrays (Affymetrix, Santa Clara, CA, USA). Total RNA extraction was carried out using the RNeasy Micro Kit (Qiagen, Germany) according to the manufacturer’s protocol and then quantified (Nanodrop). RNA quality was assessed using the RNA 6000 Nano Assay with the 2100 Bioanalyzer (Agilent, Santa Clara, CA, USA). Samples for the Gene 2.0 arrays were prepared and hybridized to the arrays according to the Affymetrix WT Plus Kit manual. Briefly, for each sample, 100 ng of total RNA was reverse-transcribed into cDNA using a random hexamer oligonucleotide tagged with a T7 promoter sequence. After second-strand synthesis, double-strand cDNA was used as a template for amplification with T7 RNA polymerase to obtain antisense cRNA. Random hexamers and dNTPs spiked out with dUTP were then used to reverse-transcribe the cRNA into single-stranded sense-strand cDNA. The cDNA was then fragmented with uracil DNA glycosylase and apurinic/apyrimidic endonuclease 1. The fragment size was checked using the 2100 Bioanalyzer and ranged from 50 to 200 bp. Fragmented sense cDNA was biotin-end-labeled with TdT and the probes were hybridized to the Gene 2.0 arrays at 45°C for 16h with 60 rpms. Hybridized arrays were washed and stained on a Fluidics Station 450 (program: FS450 0002) and scanned on a GeneChip^®^ Scanner 3000 7G (both Affymetrix). Raw image data were analyzed with Affymetrix^®^ Expression Console™ Software (Affymetrix, USA), and gene expression intensities were normalized and summarized with a robust multiarray average algorithm (23). Transcripts that were expressed differently more than 1.5-fold with a raw p-value lower than 0.05 between the sample groups were categorized as regulated. A enrichment analysis for Wiki pathways was performed using WebGestalt (24). For the enrichment analysis only, genes that changed at least 1.5-fold with a p-value lower than 0.05 between *Foxp3^cre^CREB^fl/fl^* and *CREB^fl/fl^* samples were taken into consideration.

### 2.11 TSDR methylation analysis

For all the methylation analyses, cells from male mice were used. Genomic DNA was prepared from FACS-sorted T_conv_ cells (CD4^+^CD25^-^) and T_reg_ cells (CD4^+^CD25^+^Nrp1^+^) from *CREB^fl/fl^* and *Foxp3^cre^CREB^fl/fl^* mice and subsequently converted by bisulfite according to the manufacturer’s instructions (DNeasy Blood & Tissue Kit, Qiagen; EZ DNA Methylation-Lightning Kit, Zymo Research, USA). Pyrosequencing of the Treg cell-specific demethylated region (TSDR) (chromosome position X:7583950-7584149, genome assembly GRCm38.p6) was performed as described previously (25).

### 2.12 Transposase-accessible chromatin assay using sequencing

Omni-transposase-accessible chromatin assay using sequencing (ATAC-seq) was performed according to (26, 27) with minor modifications. Prior to transposition, dead cells were removed by centrifugation (800 rpm, 4 min, 4°C). The transposition reaction was conducted with 7.5 μL Tagment DNA Enzyme 1 (TDE1) for 60 min at 37°C. Pre-amplification was conducted with NEBNext Ultra II Q5 Master Mix and Nextera PCR Primers (5 cycles). Quantitative PCR amplification was conducted with NEBNext Ultra II Q5 Master Mix, Nextera PCR Primer, and SYBR Gold to determine the number of additional cycles. PCR amplification of additional cycles was the same as for pre-amplification. PCR fragments were purified with Qiagen MinElute PCR Purification Kit and library concentration and quality were determined using an Agilent High Sensitive DNA Kit and TapeStation, respectively. ATACseq libraries were sequenced on the Illumina NextSeq 500 Platform with 75 bps paired-end reads in duplicate. ATAC-seq libraries were trimmed with Trim Galore (parameters -q 30 –paired –trim1) and aligned to the mouse genome (mm9) using Bowtie2 (28) (parameters -X2000 –no-mixed –no-discordant). Duplicate fragments were removed, and reads were filtered for alignment quality of >Q30 using samtools (29). Next, we used MAC2 (30) to perform peak calling (parameters: nomodel, nolambda, keep-dup auto, call-summits). Transcription factor footprinting and differential activity was performed using HINT-ATAC as described in Li et al. (27).

### 2.13 ELISA

Total IgG and IgE were measured from sera using the Ready-Set-Go ELISA system (affymetrix: eBioscience, USA). IL-10 was determined in cell supernatants using mouse IL-10 ELISA Ready-SET-GO! (2nd Generation) (Thermo Fisher Scientific, USA), according to the manufacturer’s instructions.

### 2.14 Statistical analysis

All data are presented as mean ± standard error (SEM). Data were tested for normality using the Shapiro–Wilk normality test. Differences between two groups were evaluated using two-tailed unpaired or paired (if indicated) Student’s t-tests. A two-tailed Mann–Whitney test was used if data were not normally distributed. One-way ANOVA was performed if there are more than two groups. Measurements were taken from distinct samples. All statistical analysis and subsequent graphics generation were performed using GraphPad Prism versions 7.0 and 8.0 (GraphPad Software, USA). A *p*-value <0.05 was statistically significant.

### 2.15 Study approval

The animal study was approved by the regional government authorities and animal procedures were performed according to German legislation for animal protection. Permission for the projects was granted by the Regierungspräsident/LANUV Nordrhein-Westfalen (81-02.04. 2017.A393).

### 2.16 Data availability

The ATAC-Seq and microarray raw datasets have been deposited in GEO under the accession codes GSE157693 and GSE157933. All data that support the findings of this study are available within the article and the **Supplementary Material**. All other data, including raw data used in each figure, will be provided upon reasonable request to the corresponding author.

## 3 Results

### 3.1 CREB enhances T_reg_ numbers but downregulates Foxp3 expression in T_regs_

**Figure 1:**
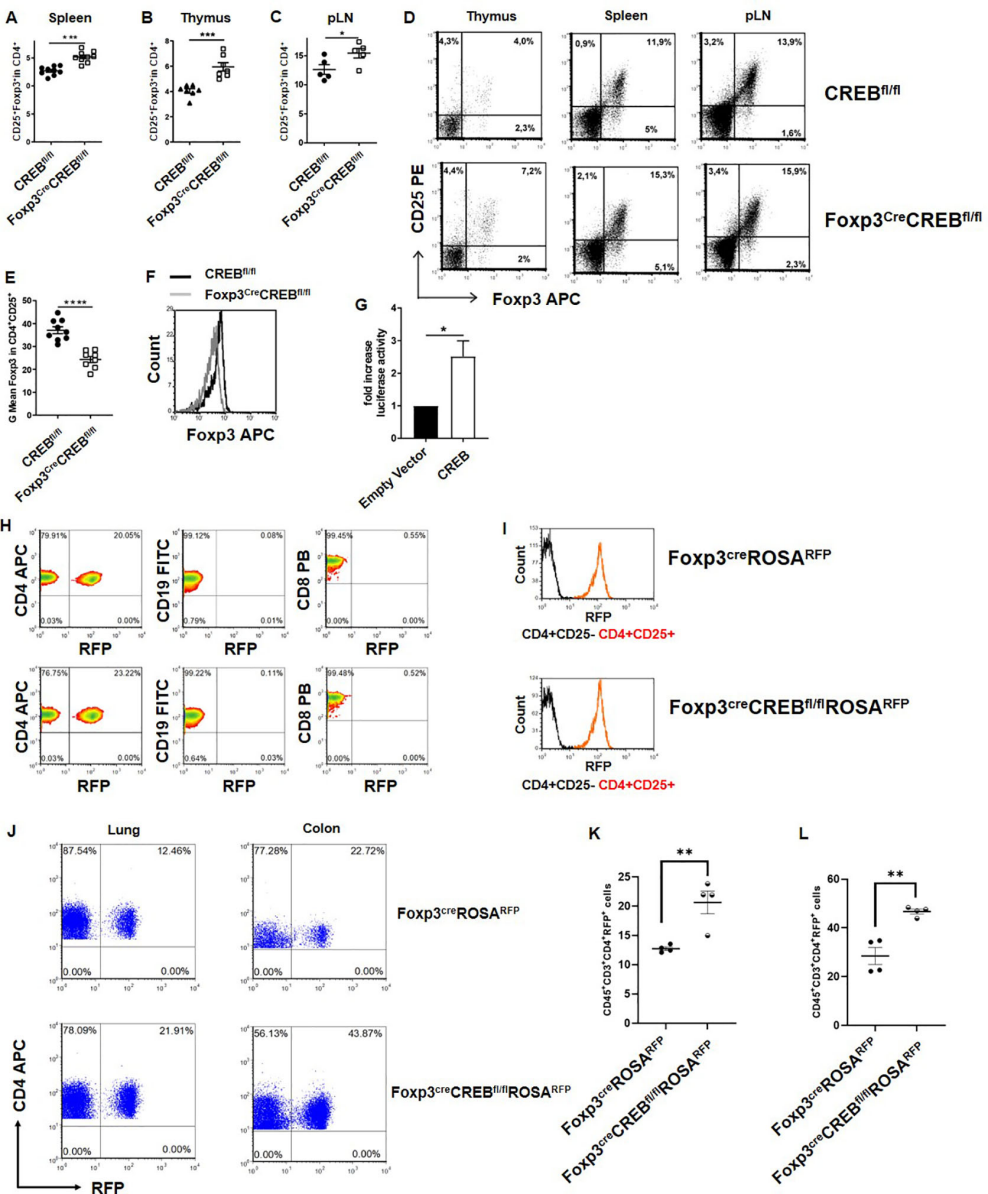
Genetic deletion of CREB in Foxp3^+^ cells increases the percentage of T_regs_ but downregulates their Foxp3 expression. **(A)** Statistical analysis of splenic T_reg_ (CD4^+^CD25^+^Foxp3^+^) percentages from *CREB^fl/fl^* and *Foxp3^cre^CREB^fl/fl^* mice (*N* = 9, four independently performed experiments, each with 2–3 age and sex-matched mice that were 6–9 weeks old). **(B)** Statistical analysis of T_reg_ percentages within the thymuses (CD3^+^CD4^sp^CD25^+^Foxp3^+^) of *CREB^fl/fl^* and *Foxp3^cre^CREB^fl/f l^* mice (*N* = 7, three independently performed experiments, each with 2–3 age and sex-matched mice that were 6–9 weeks old). **(C)** Statistical analysis of T_reg_ (CD4^+^CD25^+^Foxp3^+^) percentages from pLNs of *CREB^fl/fl^* and *Foxp3^CRE^CREB^fl/fl^* mice (*N* =5, two independently performed experiments, each with 2–3 age and sex-matched mice that were 7–9 weeks old). **(D)** Density plot showing CD25^+^Foxp3^+^ cells gated on CD3^+^CD4^+^CD8^-^ cells in the thymuses of *CREB^fl/fl^* and *Foxp3^cre^CREB^fl/fl^* mice (left), CD25^+^Foxp3^+^ cells gated on CD4^+^ cells in the spleens of *CREB^fl/fl^* and *Foxp3^cre^CREB^fl/fl^* mice (middle), and CD4^+^CD25^+^Foxp3+ cells in the pLNs from *CREB^fl/fl^* and *Foxp3^cre^CREB^fl/fl^* mice (right). **(E)** Statistical analysis of mean fluorescent intensity (MFI) of Foxp3 in *CREB^fl/fl^* and *Foxp3^cre^CREB^fl/fl^* CD4^+^CD25^+^ T cells (*N=6*, 3 independently performed experiments, each with two animals that were 6–8 weeks old and sex- and age-matched). **(F)** Representative histogram showing an overlay of Foxp3 expression in CD4^+^CD25^+^ cells from *CREB^fl/f l^* (black) and *Foxp3^cre^CREB^fl/fl^* (grey) spleens. **(G)** RLM cells carrying a stable pGL3-TSDR-FoxPro luciferase plasmid were transfected either with an empty vector or CREB plasmid and stimulated with PMA, and luciferase activity was measured after 4 hours (*N = 5*). **(H)** Percentages of RFP^+^ cells within the CD4^+^ (on the left), CD19^+^ (in the middle), and CD8^+^ populations (on the right) in the spleens of *Foxp3^cre^ROSA^RFP^* and *Foxp3^cre^CREB^fl/fl^ROSA^RFP^* mice. **(I)** Representative histogram showing the MFI of RFP expression in CD4^+^CD25- (black) and CD4^+^CD25^+^ cells (red) in the spleens of *Foxp3^cre^ROSA^RFP^* and *Foxp3^cre^CREB^fl/fl^ROSA^RFP^* mice. **(J)** Percentage of RFP^+^ cells within the CD45^+^CD3^+^CD4^+^ population in the lungs and colons of *Foxp3^cre^ROSA^RFP^* and *Foxp3^cre^CREB^fl/fl^ROSA^RFP^* mice. **(K)** Statistical analysis of CD45^+^CD3^+^CD4^+^RFP^+^ cells in the lung tissue of *Foxp3^CRE^ROSA^RFP^* and *Foxp3^cre^CREB^fl/fl^ROSA^RFP^* mice (*N=4*, three independently performed experiments). **(L)** Statistical analysis of CD45^+^CD3^+^CD4^+^RFP^+^ cells in the colon tissue of *Foxp3^cre^ROSA^RFP^* and *Foxp3^cre^CREB^fl/fl^ROSA^RFP^* mice (*N=4*, three independently performed experiments). Two-tailed, unpaired t-tests were used to test the significance. *p<0.05, **p<0.01, ***p<0.001, and ****p<0.0001 and the results are expressed as the mean ± SEM.

CREB is a transcriptional activator and has been found to critically stabilize Foxp3 expression (31). It is therefore widely accepted that CREB promotes FoxP3 expression in T_regs_ (32–34). To verify this hypothesis *in vivo*, we generated mice with a Foxp3-specific knockout of CREB (*Foxp3^cre^CREB^fl/fl^*). To our surprise, these mice showed enhanced numbers of CD25^+^/Foxp3^+^ positive T_regs_ in the thymus (**Figures 1A, D**), spleen (**Figures 1B, D**), and pLN (**Figures 1C, D**). Splenic T_regs_ showed no altered expression of Nrp1, PD-1, CTLA4, and ICOS (**Supplementary Figures 1A, B**). There was also no convincing different expression of T_h_ lineage transcription factors GATA3 and RORyT and T-bet in CD4^+^Foxp3^+^ cells within different organs (**Supplementary Figures 2A–E**). However, *Foxp3^cre^CREB^fl/fl^* mice displayed lower Foxp3 expression in T_regs_ on the single cell level (**Figures 1E, F**), which can be explained by the fact that CREB directly activates the TSDR (31) (**Figure 1G**). To verify Cre expression in the Foxp3-Cre mice, we crossed *Foxp3^cre^CREB^fl/fl^* mice with Cre-inducible reporter mice (*ROSA^R^* ^FP^) and examined reporter gene expression in lymphocytes. RFP expression was confined to CD4^+^CD25^+^ cells, which is important for excluding cell extrinsic side-effects (**Figures 1H, I**).

We further used *Foxp3^cre^CREB^fl/fl^ ROSA^R^* ^FP^ mice to assess the percentages of T_regs_ in non-lymphoid organs. While we did not observe differences in liver tissue, we found a particularly marked upregulation of T_reg_ percentages within the lungs and colons of *Foxp3^cre^CREB^fl/fl^ ROSA^R^* ^FP^ mice compared to *Foxp3^cre^ROSA* ^RFP^ mice (**Figures 1J–L**) and enhanced absolute numbers of T_regs_ in the colons of Foxp*3^cre^CREB^fl/fl^ ROSA^R^* ^FP^ (**Supplementary Figure 7B**). This prompted us to analyze whether CREB expression in T_regs_ differs in different tissues. We could not find an association between high tissue-dependent CREB expression and the effects of CREB deficiency on T_reg_ percentages; however, we found a high expression of CREB in murine colonic T_regs_ (**Supplementary Figure 8**).

### 3.2 CREB-deficient T_regs_ are suppressive *in vitro*

**Figure 2:**
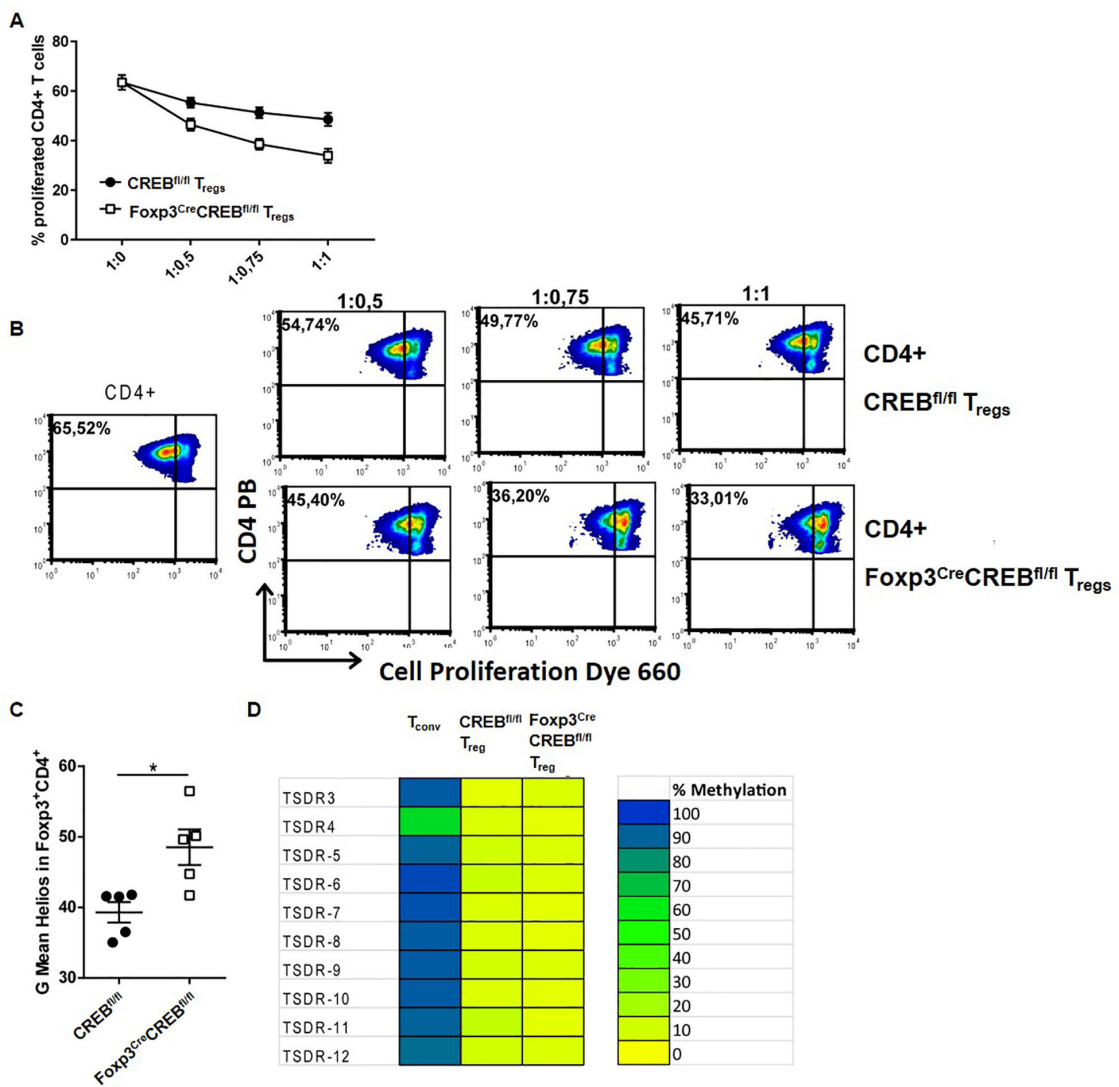
*In vitro* suppressive capacity of T_regs_ is enhanced in the absence of CREB. **(A)** WT CD4^+^ T cells from the spleens (T_eff_) were labeled with eFluor 660 and stimulated with anti-CD3/CD28 antibodies for 3 days. CD4^+^CD25^+^ T cells (T_regs_) from *CREB^fl/fl^* or *Foxp3^cre^CREB^fl/fl^* animals were added in different ratios (1:0, 1:0.5, 1:0.75, and 1:1). The proliferation of responder T cells was assessed in a eFluor 660 (eBioscience) dilution (*N = 3*) using flow cytometry **(B)** Representative density plots of **(A)**. **(C)** Statistical analysis of MFI of helios in *CREB^fl/fl^* and Foxp*3^cre^CREB^fl/fl^* CD4^+^Foxp3^+^ T cells (*N = 5*, 2 independently performed experiments, each with 2–3 animals that were 8 weeks old and sex-matched; a two-tailed Mann–Whitney test was performed to test for significance). **(D)** Representative TSDR methylation patterns of purified CD4^+^CD25^+^Nrp1^+^ T_reg_ cells and CD4^+^CD25^−^ conventional T cells isolated from the spleens of 6–8-week-old male mice (*N = 4*). Amplicons are vertically arranged, each representing a single CpG motif. The methylation rate of each motif was translated into a yellow-green-blue color code. *p<0.05, and all the results are expressed as the mean ± SEM.

To test whether lower Foxp3 expression affects the suppressive capacity of CREB-deficient T_regs,_ we co-cultured *CREB^fl/fl^* T_regs_ and *Foxp3^cre^CREB^fl/fl^* T_regs_ with anti-CD3 and anti-CD28 stimulated WT CD4^+^ cells. Surprisingly, *Foxp3^cre^CREB^fl/fl^* T_regs_ revealed an increased capacity to reduce CD4^+^ T cell proliferation (**Figures 2A, B**). These data suggest that deletion of CREB, despite reducing Foxp3 expression per cell, enhances the function of T_regs_. In addition, the expression of Helios was enhanced (**Figure 2C**) and bisulfite sequencing revealed a demethylated TSDR (**Figure 2D**), which suggests a stable and suppressive phenotype (35). In conclusion, despite the reduction of Foxp3 levels per cell, CREB deficiency leads to stable and functional T_regs_ *in vitro*.

### 3.3 CREB-deficient T_regs_ reveal gene expression signature with enhanced expression of IL-10, IL-4, IL-13, and ST2

**Figure 3:**
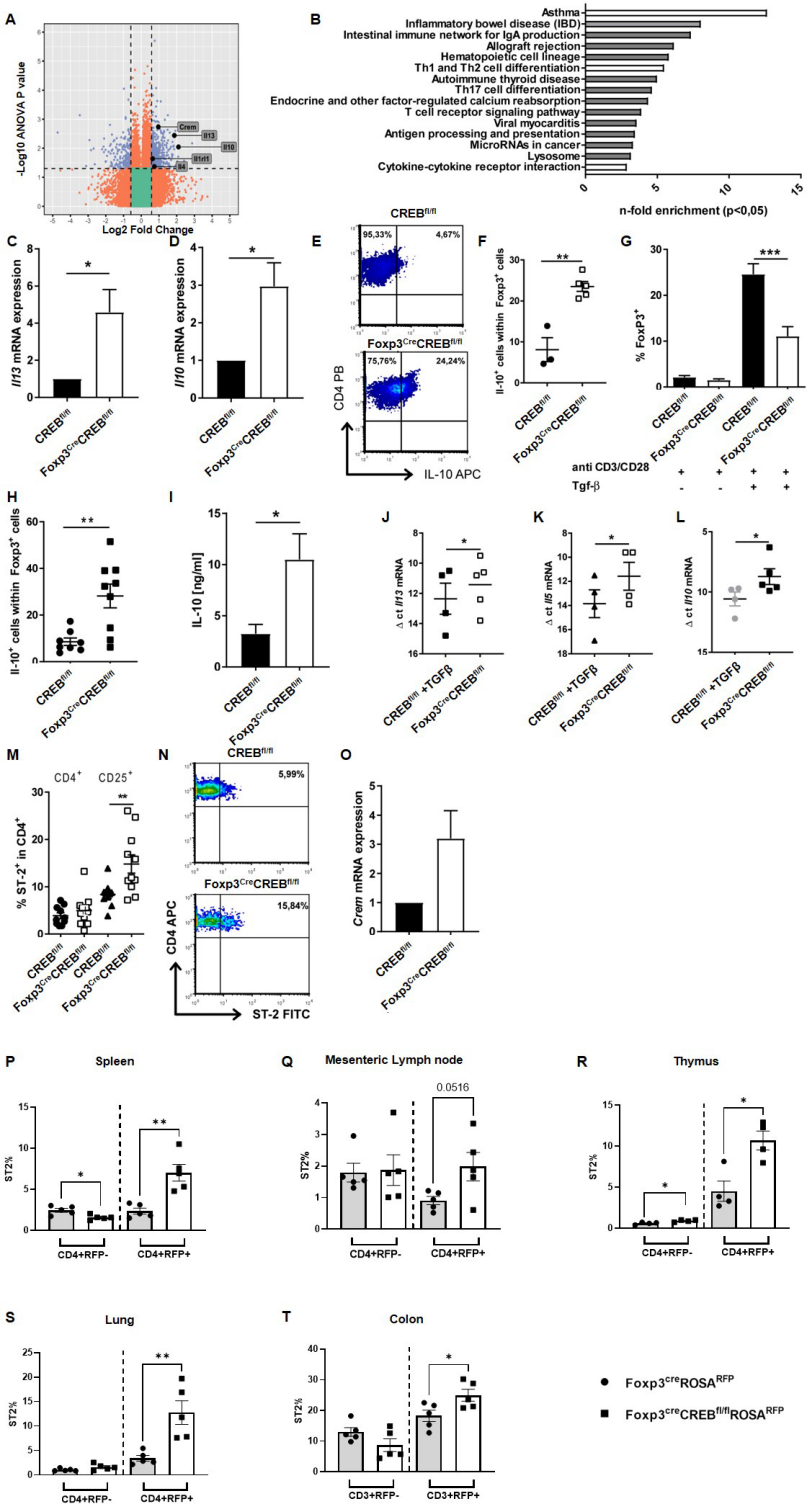
Altered gene expression in CREB deficient T_regs_. **(A)**Gene expression in T_regs_ from the spleens of *CREB^fl/fl^* and *Foxp3^cre^CREB^fl/fl^* mice. Colors indicate significant upregulation or downregulation of at least 1.5-fold with a p-value not more than 0.05 (blue); regulation of at least 1.5-fold or regulation with p-value not more than 0.05 (red). **(B)** Selection of pathways and associated genes that were significantly enriched. **(C)** N-fold *Il13* mRNA expression in splenic T_regs_ from *CREB^fl/fl^* and *Foxp3^cre^CREB^fl/fl^* mice (*N = 5*, male, 8–10 weeks old) analyzed by RT-qPCR. Bars indicate the mean and error bars the SEM, and a two-tailed one-sample test was used to test for significance. **D)** N-fold *Il10* mRNA expression in splenic T_regs_ from *CREB^fl/fl^* and *Foxp3^cre^CREB^fl/fl^* mice (*N = 5*, male, 8–10 weeks old) analyzed by RT-qPCR. Bars indicate mean and error bars SEM, and a two-tailed one-sample test was used to test for significance. **E)** MACS-isolated T cells from spleens were stimulated *ex vivo* with anti-CD3/CD28 antibodies for 3 days and re-stimulated with PMA/Ionomycin in the presence of Golgi Plug™. A representative density plot shows IL-10 production in CD4^+^Foxp3^+^ cells. **(F)** Statistical analysis of IL-10 expression of Foxp3^+^ cells after treatment as in **(E)** (*N = 3* independent experiments, each dot represents one animal, two tailed unpaired t-test. **(G)** CD4^+^CD25^-^ T cells were stimulated with anti-CD3/CD28 antibodies in the presence/absence of 5 ng/mL TGF-β for 5 days. Percentages of Foxp3^+^ cells from six independently performed experiments were determined, each with the spleen from one mouse (*N =13 CREB^fl/fl^* and *N=12 Foxp3^cre^CREB^fl/fl^* mice), and a two-tailed unpaired t-test was used to test for significance. **(H)** Statistical analysis of IL-10^+^ cells within Foxp3^+^ cells after treatment as in G). An unpaired two-tailed t-test was used to test for significance, each dot represents one animal, and error bars the SEM. **(I)** IL-10 in supernatants of CD4^+^CD25^-^ T cells after stimulation with anti-CD3/CD28 antibodies in the presence of 5 ng/mL TGF-β for 5 days was measured by ELISA, and an unpaired two- tailed t-test was performed to test for significance. The bars indicate the mean and the error bars the SEM (*N = 6*). (**J–L)** Δ ct mRNA expression of cells after treatment as in **(G)** Each dot represents one animal and error bars the SEM. Two-tailed unpaired t-tests were used to test for significance. (**M)** Statistical analysis of splenic ST2^+^ cells within CD4^+^ and CD4^+^CD25^+^ cells of *CREB^fl/fl^* and *Foxp3^cre^CREB^fl/fl^* mice. There were three independently performed experiments (*N = 10 CREB^fl/fl^* and *N =11 Foxp3^cre^CREB^fl/fl^* mice), a two-tailed unpaired t-test was used to test for significance, and each dot represents one animal and error bars the SEM. (**N)** Representative density plot of M. (**O)** N-fold *Crem* mRNA expression in splenic T_regs_ from *CREB^fl/fl^* and *Foxp3^cre^CREB^fl/fl^* mice (N = 3, male, 8**–**10 weeks old) cells analyzed by RT-qPCR. (**P–T)** Statistical analysis of ST2^+^ in CD4+ and CD4+RFP+ cells in *Foxp3^cre^ROSA^RFP^* (*N = 5*) and *Foxp3^cre^CREB^fl/fl^ROSA^RFP^* (N = 5) in the (**P)** spleens, (**Q)** mesenteric lymph nodes, (**R)** thymuses, and (**S)** lungs. Bars indicate mean and error bars the SEM, and a two-tailed unpaired t-test was used to test for significance. (**T)** Statistical analysis of ST2+ in CD3+ and CD3+RFP+ cells in *Foxp3^cre^ROSA^RFP^* (*N = 5*) and *Foxp3^cre^CREB^fl/fl^ROSA^RFP^* (*N = 5*) in the colon. A two-tailed unpaired t-test was used to test for significance. *p<0.05, **p<0.01, and ***p<0.001, and the results are expressed as the mean ± SEM.

To understand why CREB-deficient T_regs_ are suppressive despite reduced Foxp3 expression, we performed a whole transcriptome analysis (Affymetrix HTA2 arrays) of flow-sorted (CD4^+^CD25^+^Nrp-1^+^) *Foxp3^cre^CREB^fl/fl^* and *CREB^fl/fl^* T_regs_ (**Supplementary Figure 3A**) and appropriate wild-type mice because, at the time of this analysis, the *Foxp3^CRE^CREB^fl/fl^ ROSA^R^* ^FP^ mice were not yet available. Using a fold-change of 1.5 and a p-value of less than 0.05, we found 111 downregulated and 122 upregulated genes in the *Foxp3^cre^CREB^fl/fl^* compared to the *CREB^fl/fl^* T_regs_, and among them, IL-10 and IL-13 were the two most differentially regulated in the *Foxp3^cre^CREB^fl/fl^* T_regs_ (**Figure 3A**). KEGG pathway analysis revealed an enrichment of differentially regulated genes in pathways that are associated with Th2 cytokines, such as asthma, Th2 and Th2 cell differentiation, and cytokine-cytokine receptor interaction pathways (**Figure 3B**). Higher *Il13* and *Il10* mRNA expression in *Foxp3^cre^CREB^fl/fl^* T_regs_ could be further confirmed by RT-qPCR (**Figures 3C, D**). CD4^+^CD25^+^Foxp3^+^ cells also showed enhanced IL-10 protein levels after 3 days of CD3/CD28 stimulation, as assessed by flow cytometry (**Figures 3E, F**). *In vitro* T_reg_ differentiation with TGF-β revealed a reduced capacity of *Foxp3^cre^CREB^fl/fl^* T cells to differentiate towards Foxp3^+^ cells, while IL-10 expressing cells within Foxp3^+^ cells were enhanced (**Figure 3G**). Furthermore, IL-10 cytokine expression of TGF-β−stimulated *Foxp3^cre^CREB^fl/fl^* T cells measured by ELISA was enhanced, as was *Il13, Il5*, and *Il10* mRNA expression, which suggests that CREB deficiency in T_regs_ induces a Th2-biased phenotype (**Figures 3H-L**). Beyond cytokines, Il1r1 (ST2) showed enhanced expression on the microarray. ST2, the receptor of IL-33, is preferentially expressed on colonic T_regs_, where it promotes T_reg_ function and adaptation to the inflammatory environment. It provides a necessary signal for T_reg_-cell accumulation and maintenance in inflamed tissues, and it was shown to regulate IL-10-expressing T_regs_ in the gut (8). In addition, IL-33 has been identified as a major interleukin in asthma pathology (36). ST2 was highly expressed in the *Foxp3^cre^CREB^fl/fl^* T_regs_, which was confirmed by flow cytometry (**Figures 3M, N**). Moreover, the whole transcriptome analysis also revealed an upregulated expression of CREM in *Foxp3^cre^CREB^fl/fl^* T_regs_ and qRT-PCR also showed tendentially enhanced CREM expression in *Foxp3^cre^CREB^fl/fl^* T_regs_ (**Figure 3O**). In conclusion, these data suggest that CREB deletion induces ST2^+^ T_regs_. This finding led us to investigate the protein expression of ST2 within different organs in *Foxp3^cre^ROSA^RFP^* and *Foxp3^cre^CREB^fl/fl^ROSA^RFP^* mice. ST2 expression was clearly enhanced in T_regs_ in the thymus, spleens, mesenteric lymph nodes, lungs, and colons of these mice (**Figures 3P–T**).

### 3.4 *Foxp3^cre^CREB^fl/fl^* T cells dampen inflammation in experimental colitis

**Figure 4:**
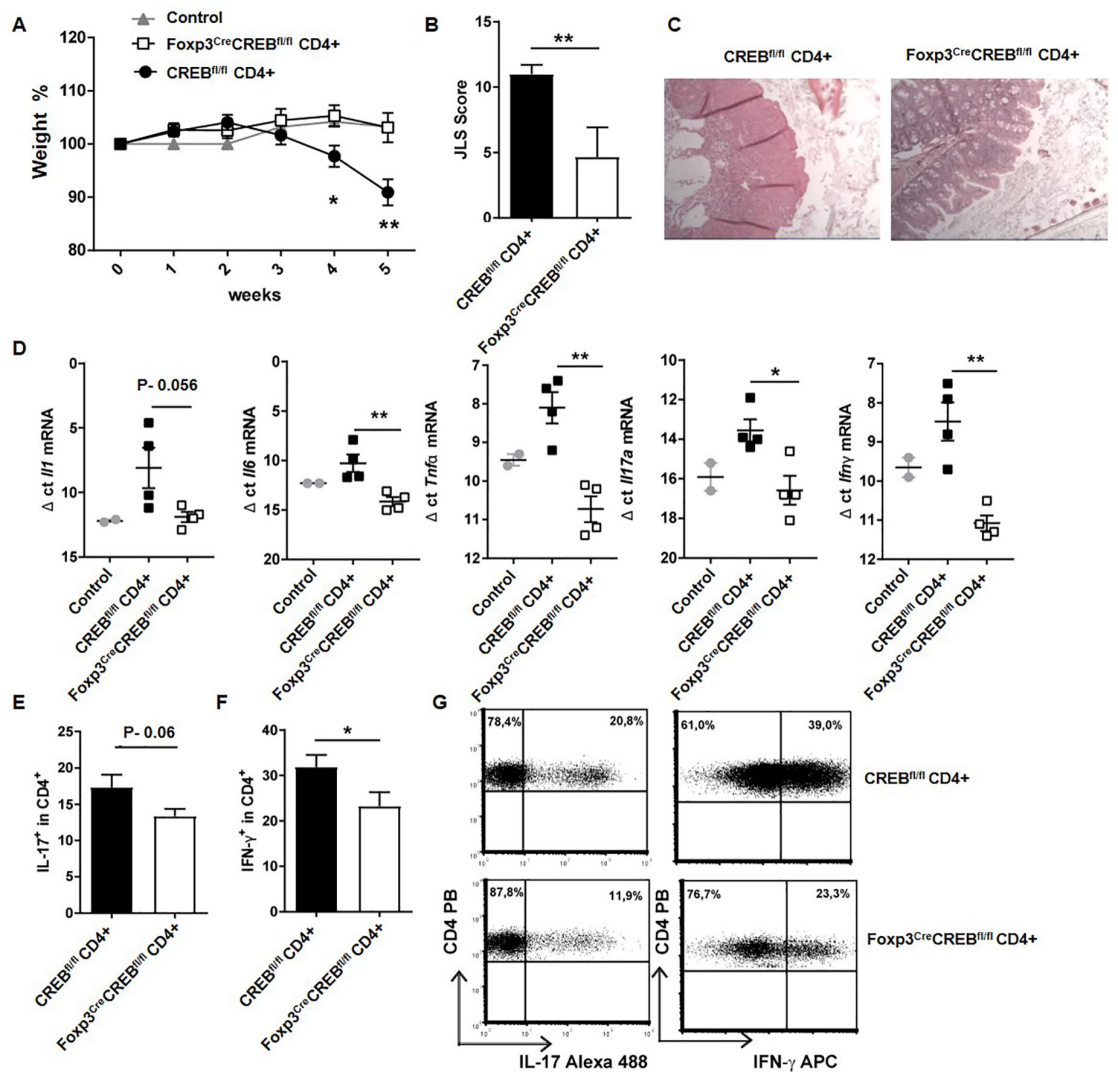
*Foxp3^cre^CREB^fl/fl^* CD4+ T cells induce only moderate inflammation in experimental colitis. Rag2^-/-^ mice were adoptively transferred CD4^+^ CD25^-^ wild-type cells (*CREB^fl/fl^* CD4^+^) or *Foxp3^cre^CREB^fl/fl^* CD4^+^ CD25^-^ T cells. Untreated Rag^-/-^ mice were used as controls. Mice were weighed and sacrificed 5 weeks after transfer. **(A)** Body weight as a percentage of starting weight. A two-tailed unpaired t-test were used to test for significance (*N = 11 CREB^fl/fl^*, *N = 9 Foxp3^cre^CREB^fl/fl^* recipients, and five control Rag^-/-^ mice were analyzed in three independently performed experiments). **(B)** Results of histological JLS score of colon sections (two independently performed experiments; *CREB^fl/fl^* recipients N = 5, *Foxp3^cre^CREB_fl/fl_* recipients N = 6; a two-tailed Mann**–**Whitney test was used). **(C)** Representative photomicrographs of H&E-stained colon sections imaged using 10x magnification. **(D)** Expression of inflammatory cytokines analyzed by RT-qPCR. Dots represent Δ ct values normalized to β-actin. Each dot represents one animal (ct levels are inversely proportional to the amount of target nucleic acid in the sample). Two-tailed unpaired t-tests were used to test for significance (*N = 2* control mice, *N = 4 CREB^fl/f^*, *N = 4 Foxp3^cre^CREB^fl/fl^* recipients). **(E)** Statistical analysis of IL-17^+^ cells within CD4^+^ cells in mLNs (three independently performed experiments, *CREB^fl/fl^* recipients N = 9, *Foxp3^cre^CREB^fl/fl^* recipients N = 9). A two-tailed unpaired t-test was used. **(F)** Statistical analysis of IFN-γ^+^ cells within CD4^+^ cells in mLNs (Three independently performed experiments; *CREB^fl/fl^* recipients *N = 11*, *Foxp3^cre^CREB^fl/fl^* recipients *N = 9*). A two-tailed unpaired t-test was used. **(G)** Representative dot plots of E and **(F)** *p<0.05 and **p<0.01 and the results are expressed as the mean ± SEM.

**Figure 5:**
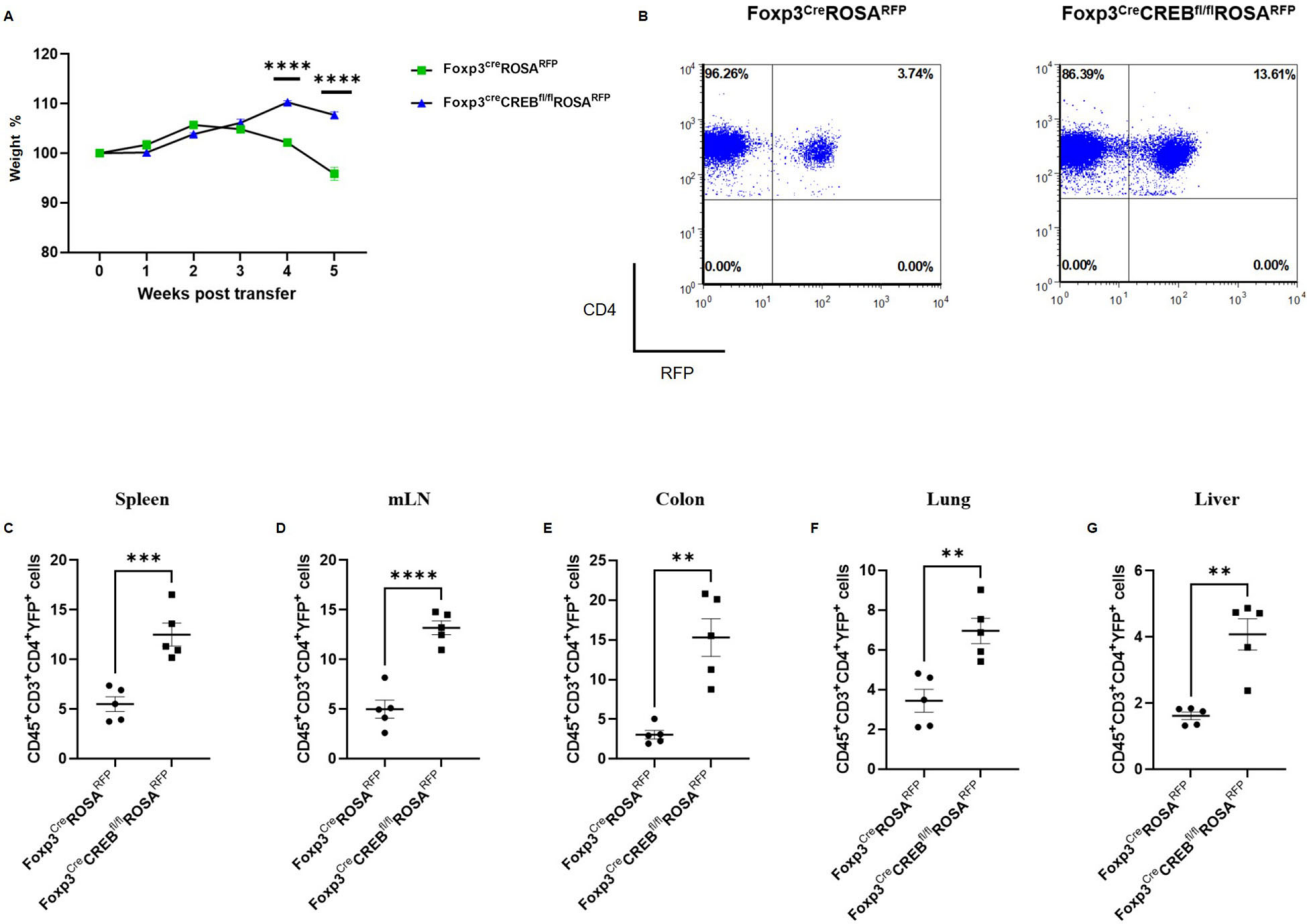
*Foxp3^cre^CREB^fl/fl^ROSA^RFP^* CD4+ T cells induce only moderate inflammation in experimental colitis, while CD4+RFP+ cells expand in peripheral tissues. Rag2^-/-^ mice were adoptively transferred CD4^+^ CD25^-^ wild-type cells (*Foxp^cre^ROSA^RFP^* CD4^+^) or *Foxp3^cre^CREB^fl/lf^ROSA^RFP^* CD4^+^ CD25^-^ T cells. Mice were weighed and sacrificed 5 weeks after transfer. **(A)** Body weight as a percentage of starting weight. A two-tailed unpaired t-test was used to test for significance (*N = 5 Foxp^cre^ROSA^RFP^* and *N = 5 Foxp3^cre^CREB^fl/lf^ROSA^RFP^* recipient mice were analyzed in one independently performed experiment). B) Dot plots representing frequencies of RFP^+^ cells within the CD45^+^CD3^+^CD4^+^ population in the spleens of Rag2-/- mice that received cells from either *Foxp3^cre^ROSA^RFP^* or *Foxp3^cre^CREB^fl/fl^ROSA^RFP^* mice. Percentages of CD45^+^CD3^+^CD4^+^RFP^+^ cells in the **(C)** spleens, **(D)** mLNs, **(E)** colons, **(F)** lungs, and **(G)** livers. A two-tailed unpaired t-test was used. **p<0.01, ***p<0.001, and ****p<0.0001 and the results are expressed as the mean ± SEM.

Since ST2^+^T_regs_ also play a protective role in intestinal mucosal inflammation (8), we performed a transfer of *Foxp3^cre^CREB^fl/fl^* T cells (CD4+CD25-) or appropriate *CREB^fl/fl^* T cells (CD4+CD25-) into lymphopenic Rag2^-/-^ mice. This usually results in the development of colitis within 5 weeks. Strikingly, the transfer of *Foxp3^cre^CREB^fl/fl^* T cells did not result in the expected weight loss (**Figure 4A**) and the recipient mice displayed decreased intestinal inflammation (**Figures 4B, C**). Furthermore, there was a significant decrease of *Il6, Tnfa, Il17a*, and *Ifng*, along with a non-significant trend towards decreased *Il1b* in the gut (**Figure 4D**) and reduced IFN-γ production. There was also a trend towards reduced IL-17A in CD4^+^ T cells in the mLNs (**Supplementary Figure S4E–G**). Limitations include the absence of IL-6 and TNFα protein data due to the limited availability of the tissue. The experiment was repeated with CD4+CD25- T cells from *Foxp3^cre^ROSA^R^* ^FP^ and *Foxp3^cre^CREB^fl/fl^ ROSA^R^* ^FP^ mice into Rag2-/- mice and with CD4+CD25- cells from *CREB^fl/fl^ and VAV^cre^CREB^fl/fl^* mice into Rag2-/- mice, with the same results. The transfer of CD4+CD25- T cells from *Foxp3^cre^CREB^fl/fl^ ROSA^R^* ^FP^ mice (**Figures 5A–G**) and from *VAV^cre^CREB^fl/fl^* prevented the development of colitis (**Supplementary Figure 11**) and resulted in enhanced accumulation of RFP+ T_regs_ in different organs (**Figures 5A–G**). Interestingly, RFP+ T_regs_ also accumulated in the liver, while we did not find a difference in the RFP expression in the *Foxp3^cre^CREB^fl/fl^ ROSA^R^* ^FP^ mice under steady state conditions.

### 3.5 Blockade of IL-10 but not of ST2 signaling reverses the protective effects of *Foxp3^cre^CREB^fl/fl^* T cells in experimental colitis

**Figure 6:**
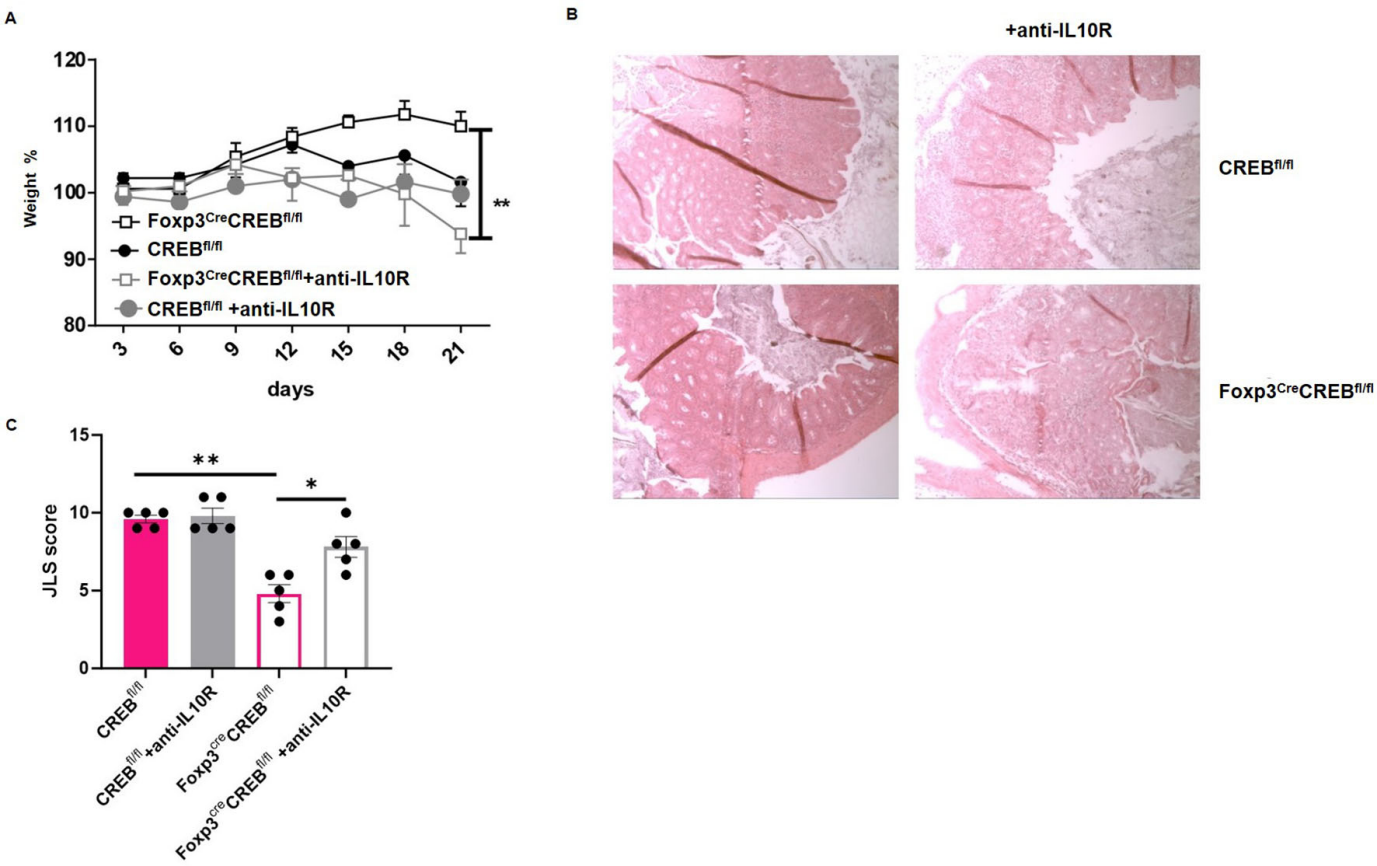
Reduced colitis in *Foxp3^cre^CREB^fl/fl^* CD4^+^ T cell recipients depends on IL-10 signaling. Rag2^-/-^ mice were adoptively transferred CD4^+^ wild-type cells (WT CD4^+^) or *Foxp3^cre^CREB^fl/fl^* CD4^+^ T cells. Mice were either treated with an anti-IL10R antibody or an isotype control. **(A)** Body weight as a percentage of starting weight. Bars indicate mean and error bars the SEM (*N=5* animals in each group; a two-tailed unpaired t-test was used; two independently performed experiments). **(B)** Representative photomicrographs of a H&E-stained colon section from *CREB^fl/fl^* and *Foxp3^cre^CREB^fl/fl^* CD4^+^ T cell recipients that were treated with anti-IL10R, imaged using 10x magnification. **(C)** Results of histological JLS score of colon sections (two independently performed experiments with overall *N=5* mice in each group; a two-tailed Mann**–**Whitney test was used to test for significance). *p<0.05 and **p<0.01 and the results are expressed as the mean ± SEM.

ST2^+^ T_regs_ are protective in intestinal inflammation (8), and,in addition, they are Th_2_ biased and release IL-10 (37). To decipher whether *Foxp3^cre^CREB^fl/fl^* T cells prevent colitis through IL-10 production and/or ST2 expression, we performed an adoptive transfer colitis experiment with anti-IL10R antibodies or recombinant soluble ST2 (sST2). sST2 acts as a decoy for IL-33 and inhibits its biological activity (38). The blockade of IL-10 signaling induced colitis in the formerly resistant *Foxp3^cre^CREB^fl/fl^* T cell-recipient mice (**Figures 6A–C**), while the ST2 blockade had no effect on disease progression (**Supplementary Figure 4**).

### 3.6 Genome-wide chromatin accessibility of WT and CREB-deficient T_regs_

**Figure 7:**
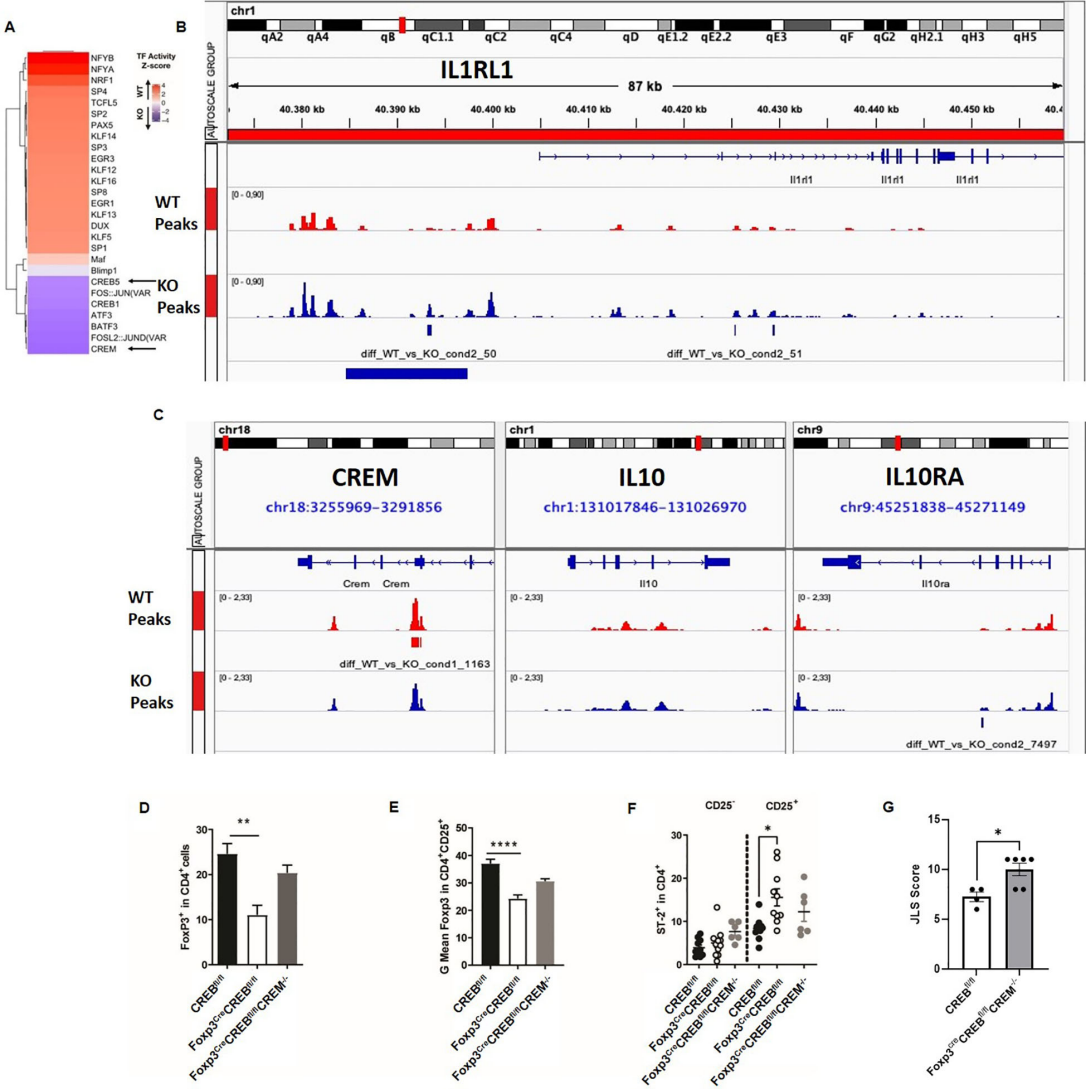
Genome-wide chromatin accessibility of *CREB^fl/fl^* and *Foxp3^cre^CREB^fl/fl^* mice. **(A)** ATAC-seq foot printing analysis indicates a decrease in TF activity for CREB and CREM upon CREB KO and an increase in TF activity of NRF1 upon CREB KO (p-value < 0.05; z-test). **(B, C)** Example of genes with loss of chromatin accessibility upon CREB KO includes enhancer regions around SC-2/Il1rl1 (blue peaks) and an alternative promoter of CREM (red peaks) (adjusted p-value < 0.05; MACS2 diff. peak caller). **(D)** CD4^+^CD25^-^ T cells were stimulated with anti-CD3/CD28 antibodies in the presence/absence of 5 ng/mL TGF-β for 5 days. Percentages of Foxp3^+^ cells from six independently performed experiments were determined, each with the spleen from one mouse (*N* = 13 *CREB^fl/fl^*, *N = 12 Foxp3^cre^CREB^fl/fl^*, and *N = 3 Foxp3^cre^CREB^fl/fl^CREM* ^-/-^ mice; a two-tailed unpaired t-test was used to test for significance; bars indicate the mean and error bars the SEM). **(E)** Statistical analysis of MFI of Foxp3 in *CREB^fl/fl^* (*N* = 13), *Foxp3^cre^CREB^fl/fl^* (N = 9), and *Foxp3^cre^CREB^fl/fl^CREM* ^-/-^ (*N* =3) CD4^+^CD25^+^ T cells. Bars indicate the mean and error bars the SEM, and a two-tailed unpaired t-test was used to test for significance. **(F)** Statistical analysis of ST2^+^ in CD4^+^ and CD4^+^CD25^+^ cells in *CREB^fl/fl^* (*N* = 5), *Foxp3^cre^CREB^fl/fl^* (*N = 6*), and *Foxp3^cre^CREB^fl/fl^CREM* ^-/-^ (*N* =3) CD4^+^CD25^+^ T cells. Bars indicate the mean and error bars the SEM, and one-way ANOVA was used to test for significance. **(G)** Rag2^-/-^ mice were adoptively transferred *CREB^fl/fl^* CD4 T-cells (CD4^+^CD25-) or *Foxp3^cre^CREB^fl/fl^* CREM^-/-^ CD4^+^ T cells (CD4+CD25-). Histological JLS score results of the colon sections (one experiment with overall *N=4/5* mice in each group; a two-tailed Mann**–**Whitney test was used to test for significance). *p<0.05, **p<0.01, and ****p<0.0001 and the results are expressed as the mean ± SEM.

To analyze whether CREB deletion alters chromatin accessibility at different promoters and enhancers, we performed ATAC-seq in *CREB^fl/fl^* and *Foxp3^cre^CREB^fl/fl^* T_regs_. We observed a decrease in open chromatin around CREB and CREM motifs in the *Foxp3^cre^CREB^fl/fl^* mice, which indicates loss of binding activity of these transcription factors in these cells (**Figure 7A**). The *Il1rl1* (ST2) region showed significant differences in open chromatin around the original and an alternative promoter in T_regs_ from the *Foxp3^cre^CREB^fl/fl^* mice, while canonical CRE motifs, which are 8-base pair palindromic sequences (5′-TGACGTCA-3′), could not be identified (**Figure 7B**). In addition, the IL-10 enhancer region showed clear differences between the two mouse strains. Within this region, binding sites for Nrf1 were identified, which is a CREB-dependent transcription factor (39). Moreover, we found a change in open chromatin of an alternate promoter of CREM (**Figure 7C**). CREM has been identified as one of the most regulated genes in ST-2 positive colonic T_regs_ of healthy mice (40). To prove whether CREM is involved in the regulation of ST2, we generated *Foxp3^cre^CREB^fl/fl^CREM^-/-^* mice. *In vitro* differentiation towards Foxp3+ cells was not significantly diminished in *Foxp3^cre^CREB^fl/fl^CREM^-/-^* mice (**Figure 7D**). Furthermore, deletion of CREM normalized Foxp3 expression per cell in CREB-deficient T_regs_ at least to some extent (**Figure 7E**) but reversed the expression of ST2 (**Figure 7F**). Taken together, our data suggest that the interaction of CREB and CREM determines the expression of Foxp3 and ST2 and the induction of CREM in CREB-deficient T_regs_ contributes to reduced Foxp3 expression. To prove the effect *in vivo*, we performed a transfer of *Foxp3^cre^CREB^fl/fl^* CREM^-/-^ T cells (CD4+CD25-) or appropriate wild-type T cells (CD4+CD25-) into lymphopenic Rag2^-/-^ mice. The additional genetic deletion of CREM reversed the effect of the CREB deletion and resulted in enhanced colitis severity, measured by JLS (The Jackson Laboratory Scoring) score in the transferred mice (**Figure 7G**).

## 4 Discussion

In this study, we provide evidence that CREB cells intrinsically regulate Foxp3 expression in T_regs_ and thereby play a critical role in T_reg_ cell-mediated immune homeostasis. In detail, CREB deficient T_regs_ showed a reduced expression of Foxp3 per cell but enhanced expression of ST2, IL-10, and IL-13. Enhanced IL-10 expression of CREB-deficient T_regs_ prevents T cell-mediated colitis. Enhanced IL-10 expression is also a hallmark of ST2^+^ T_regs_. ST2 is expressed on colonic T_regs_ and enhances local intestinal T_reg_ cell responses (8). We identified particularly enhanced expression of T_regs_ in the colons and lungs of *Foxp3^cre^CREB^fl/fl^* mice, which also includes upregulation of ST2 expression and may contribute to the unexpectedly protective role of CREB deletion in T_regs_ in type I immune responses. This protective role is in line with Wang et al. (19) who demonstrated an important role for CREB in regulating the balance between Th17 cells and T_regs_. They showed that mice bearing a deletion of CREB in all CD4^+^ T cells prevented T cell-mediated transfer colitis comparable to our model. Moreover, these mice were also resistant in an EAE model. This protective effect in EAE was surprisingly lost when CREB was specifically deleted in T_regs_. Nevertheless, except in the EAE model, Wang et al. did not analyze the Foxp3-specific CREB-deleted mice in further detail.

We hypothesize that, in particular, the enhanced IL-10 secretion prevented colitis in Rag-/- mice that received *Foxp3^cre^CREB^fl/fl^* CD4+ T cells. IL-10 is an important regulator of intestinal homeostasis, as IL-10 and IL-10R deficient mice spontaneously develop intestinal inflammation (41, 42) and blocking IL-10 signaling induced colitis in *Foxp3^cre^CREB^fl/fl^* T cell recipients that were previously resistant. It has been shown before that CREB induces IL-10 transcription in macrophages. Our data, thus, were highly unexpected and do not support these findings but may instead argue for cell-dependent mechanisms that regulate IL-10 expression. Our ATAC-seq data however do not show a direct effect of CREB expression on chromatin accessibility within the IL-10 enhancer and promoter region, thus we currently cannot mechanistically explain these findings.

We also found enhanced percentages of T_regs_ within the thymus, spleens, and pLNs but reduced Foxp3 expression per cell within peripheral lymphoid organs. Wang et al. also suggest that compensation by related transcription factors such as CREM exist. Interestingly and corroborating that theory, we found a clear upregulation of CREM in CREB-deficient T_regs_ and additional deletion of CREM recuperates Foxp3 expression in CREB deficient T_reg_ cells, while it downregulates ST2 expression. Strikingly, CREM expression, which was enhanced in *Foxp3^cre^CREB^fl/fl^* T_regs_, was found before as a highly regulated gene in ST2-positive colon and skin T_regs_ (40). Delacher et al. characterized ST2^+^ tissue T_regs_ that are present in all non-lymphoid tissues, which were found to express killer cell lectin-like receptor subfamily G1 (KLRG1) and Th2-associated factors, including IL-10 (11, 12). These “tisT_regs_ST-2” cells are programmed by BATF (12) in lymphoid organs and perform important tissue homeostasis and regenerative functions. Our data point to the fact that the expression of CREB and CREM also regulate these tisT_reg_ST2 in non-lymphoid tissues such as the colon, lungs, and liver. Spath et al. vigorously analyzed ST2 trajectories in mice and showed a high plasticity in ST2 expression and hierarchies in tissue-specific phenotypes (43). In addition, they found that ST2^+^ T_regs_ were highly proliferative and had a high migratory potential. This is in line with our findings that these cells can be found in the spleen, lung, liver, mLN, and the colon. They additionally found a high prevalence in skin and VAT, which was not analyzed in our setting. When comparing the transcriptional profiles with our analyses, we can confirm an upregulation of PTPN13 in our data (1.97-fold) and a regulation of Rab4a (-1.51-fold) between the CREB-deficient T_regs_ and wild-type T_regs_, and the other factors that distinguished ST2^+^ from ST2^-^ T_regs_ (Lyn, Gata3, Rln3, Klrg1, and Tbc1d4) were not different. Interestingly, in their analysis, ST2^+^ T_regs_ were enriched in more activated, differentiated T_reg_ populations, such as ID2^+^ T_regs_, and ID2 was upregulated in our analysis as well (1.7-fold) (43).

The limitations of our study are that we mainly mechanistically analyzed splenic T_regs_, while ST2 is a hallmark of tissue T_regs_, and further analysis will show how this affects local T_regs_ and tissue homeostasis. We observed an increase in regulatory T cell (Treg) frequencies and elevated ST2 expression in both lung and colon mucosal tissues. However, a limitation of our current study is that we did not perform comprehensive epigenetic and transcriptional profiling of the T_regs_ from these tissues, nor did we investigate the role of CREM, which has been suggested to compensate for ST2 expression from the splenic T_regs_. These important aspects will be addressed in future studies to provide a deeper understanding of the mechanisms underlying T_reg_ function and regulation in mucosal environments. Another issue is that it is not clear how CREB activation is regulated in T_regs_. CREB has nine serine residues in the kinase inducible domain (KID) that can be phosphorylated and activated by different kinases and different phosphorylation patterns of CREB can exert opposite effects. Further research will therefore be necessary to identify therapeutic targets to directly influence CREB activity in T_regs_. In conclusion, we provide evidence that CREB plays a pivotal role in T_regs_. CREB is an important transcription factor to maintain Foxp3 expression, and the interaction of CREM and CREB is crucial for the expression of ST2 in T_regs_. A lack of CREB enhances IL-10 expression and thereby prevents Th1-mediated diseases.

## Data Availability

The datasets presented in this study can be found in online repositories. The names of the repository/repositories and accession number(s) can be found below: https://www.ncbi.nlm.nih.gov/geo/, GSE157693 https://www.ncbi.nlm.nih.gov/geo/, GSE157933.
