# Supplementary material for: CREB regulates Foxp3+ST-2+ TREGS with enhanced IL-10 production
Source: Front Immunol. 2025 Jul 24;16:1601008. doi: 10.3389/fimmu.2025.1601008 (PMC12328323; doi:10.3389/fimmu.2025.1601008)
Supplement: Supplementary file 1 [file Table1.docx]

**APPENDIX B**

**SUPPLEMENTARY FIGURES**


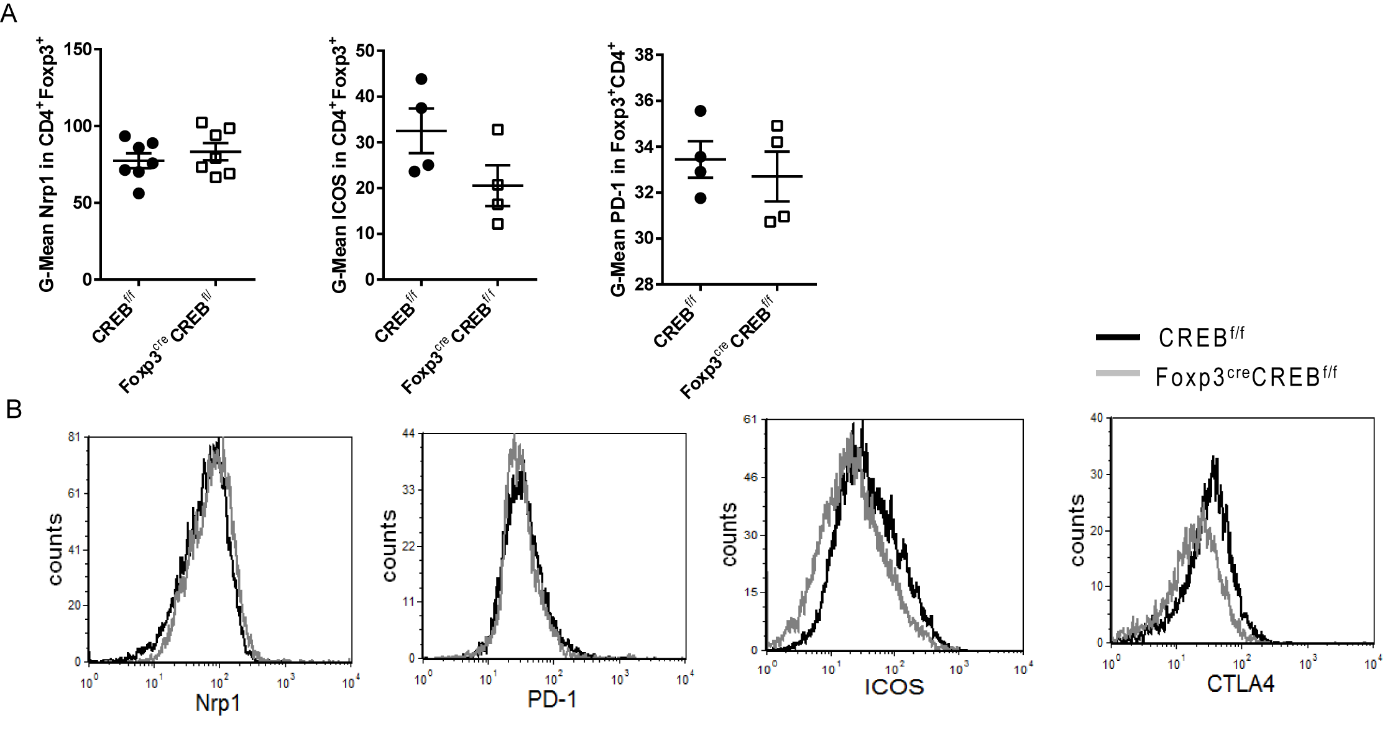


**Supplementary Figure 1:** **Characterization of CREB-deficient T_reg_ cells.** **A)** Statistical analysis and **B)** representative histograms of T_reg_ cell markers in CD4^+^Foxp3^+^ splenic cells. Mice were 6-9 weeks old, and sex and age matched. For A, results are expressed as the mean ± SEM.


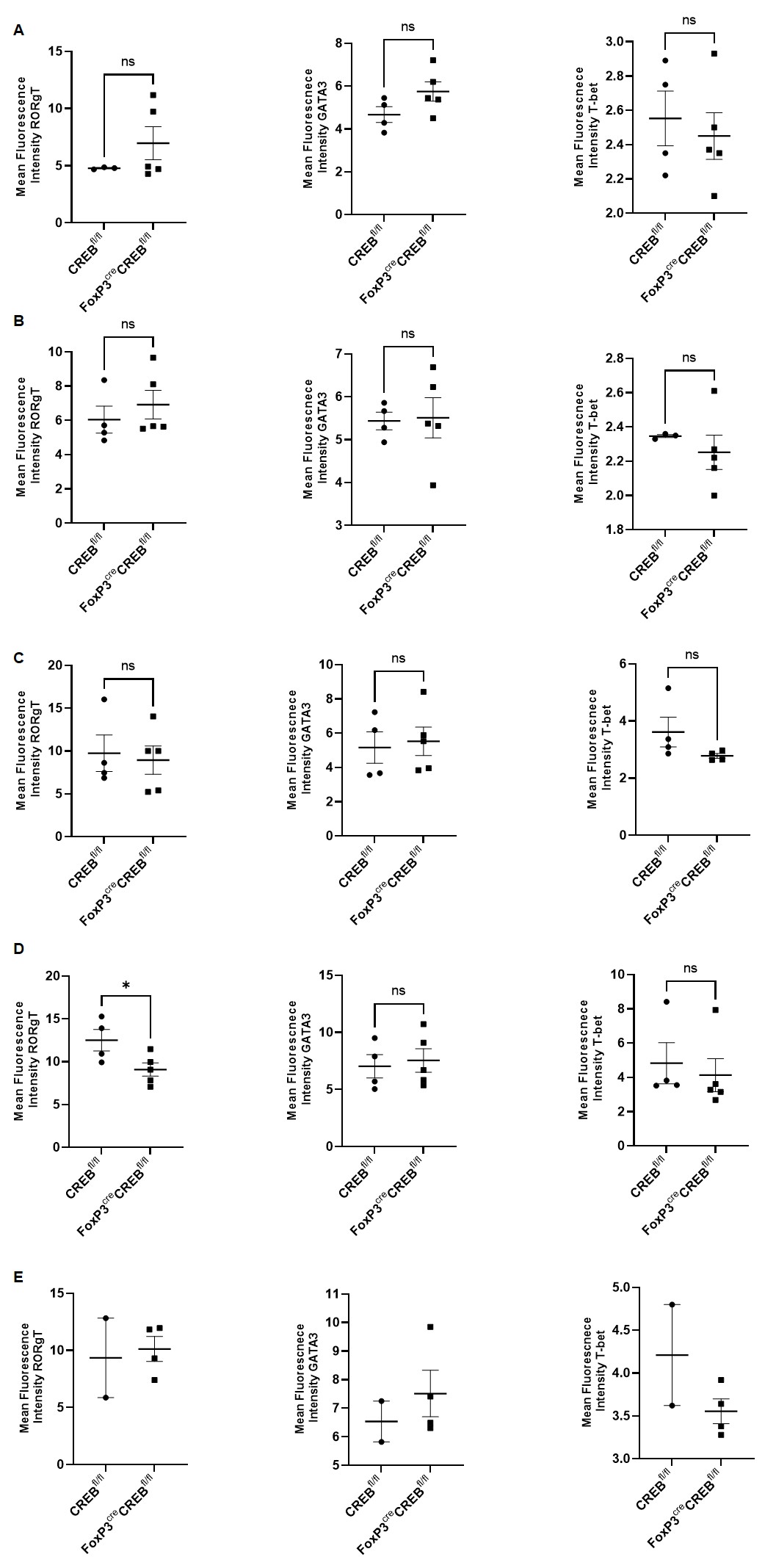


**Supplementary Figure 2: Expression of T_h_-lineage transcription factors in different organs.** Mean-fluorescent intensity of RORγt, GATA3 and T-bet expression in CD45^+^CD4^+^Foxp3^+^ cells in **A)** Spleen, **B)** Mesenteric lymph node, **C)** Lung, **D)** Liver, and **E)** Colon. Each dot represents one animal. ns - p>0.05, *p<0.05 and results are expressed as the mean ± SEM.


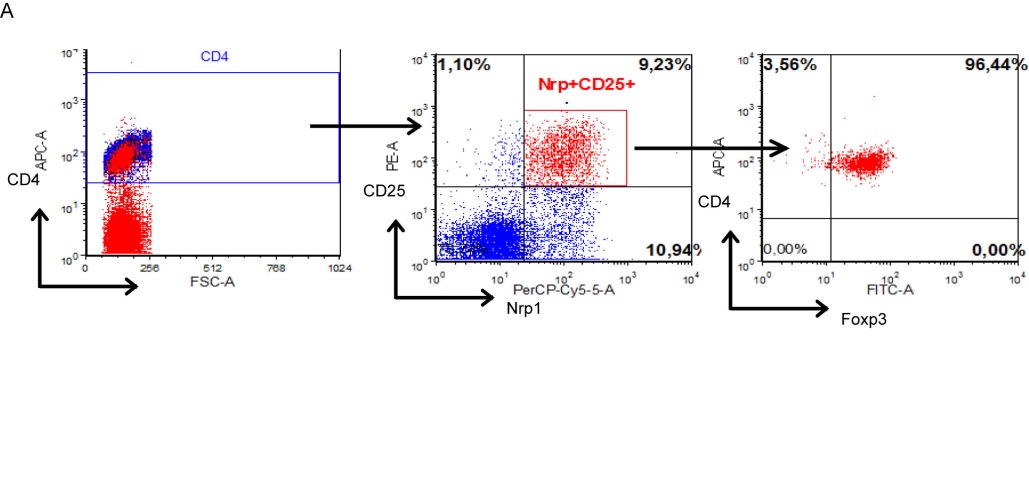


**Supplementary Figure 3: Gating strategy to sort T_reg_ cells.** We sorted Nrp1^+^CD25^+^CD4^+^ cells and thereby achieved a purity of 96% Foxp3^+^ cells.


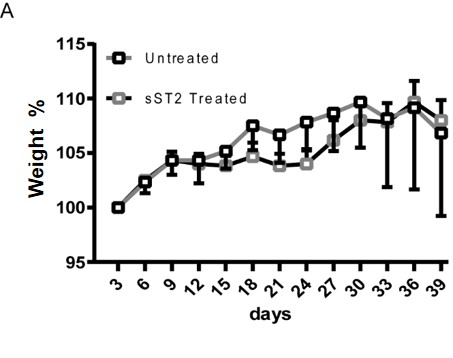


**Supplementary Figure 4: Reduced colitis in *Foxp3^cre^CREB^fl/fl^* CD4^+^ T cell recipients is unaffected by ST2 blockade.** **A)** Rag2^-/-^ mice were adoptively transferred with *Foxp3^cre^CREB^fl/fl^* CD4^+^ T cells (CD4+CD25-). Mice were either treated with sST2 or PBS. Body weight is shown as percentage of starting weight (*N = 6*, symbols indicate mean and error bars represent means ±SEM).

**
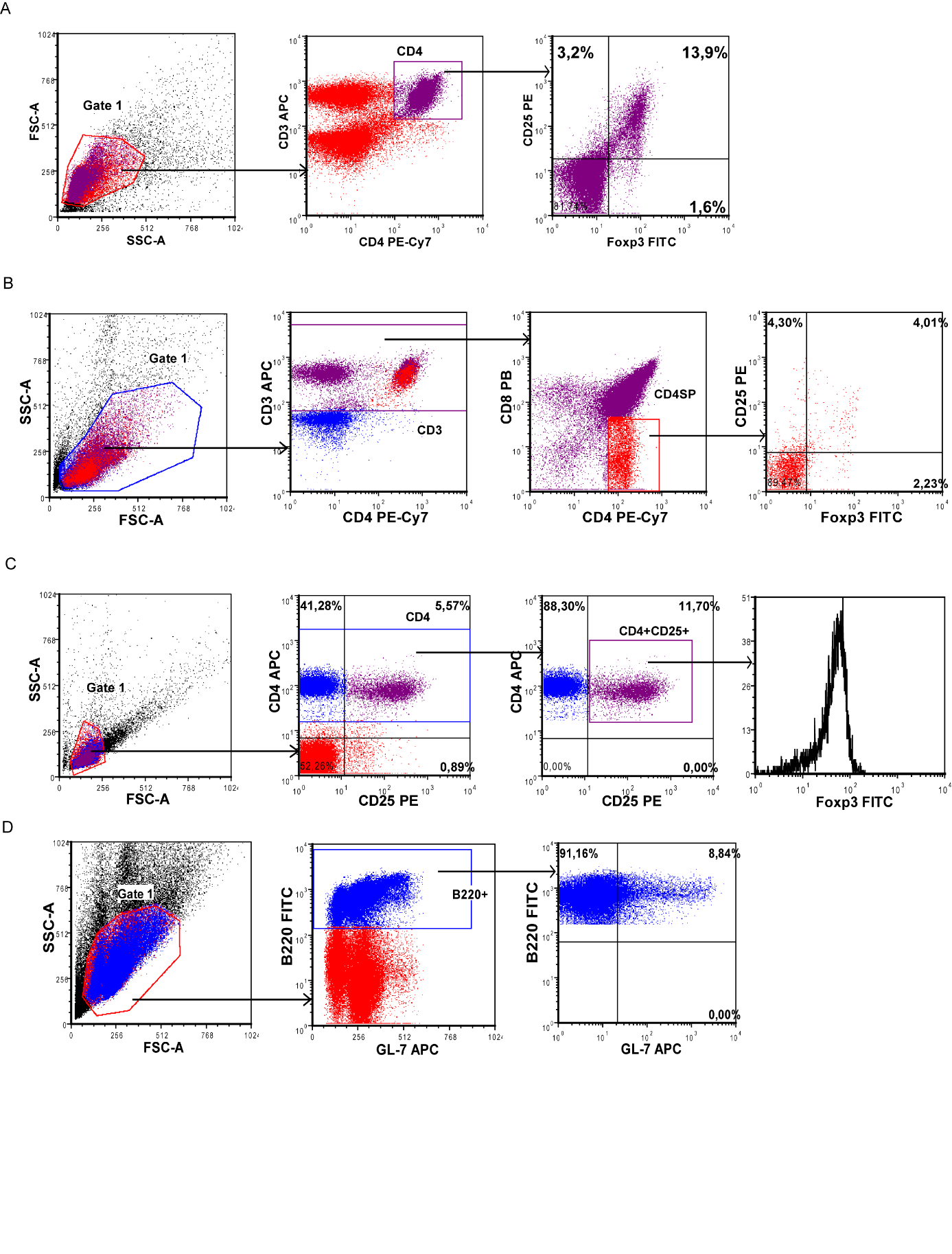
**

**Supplementary Figure 5: Gating strategies. A)** Figure exemplifying gating strategy to determine CD25^+^Foxp3^+^ T_regs_ within LNs and spleens. **B)** Figure exemplifying gating strategy to determine CD25^+^Foxp3^+^ T_regs_ within thymus. **C)** Figure exemplifying gating strategy to determine Foxp3 MFI within CD4^+^CD25^+^ cells. **D)** Figure exemplifying gating strategy to determine percentages of GL-7^+^ cells within B220^+^ cells.

**
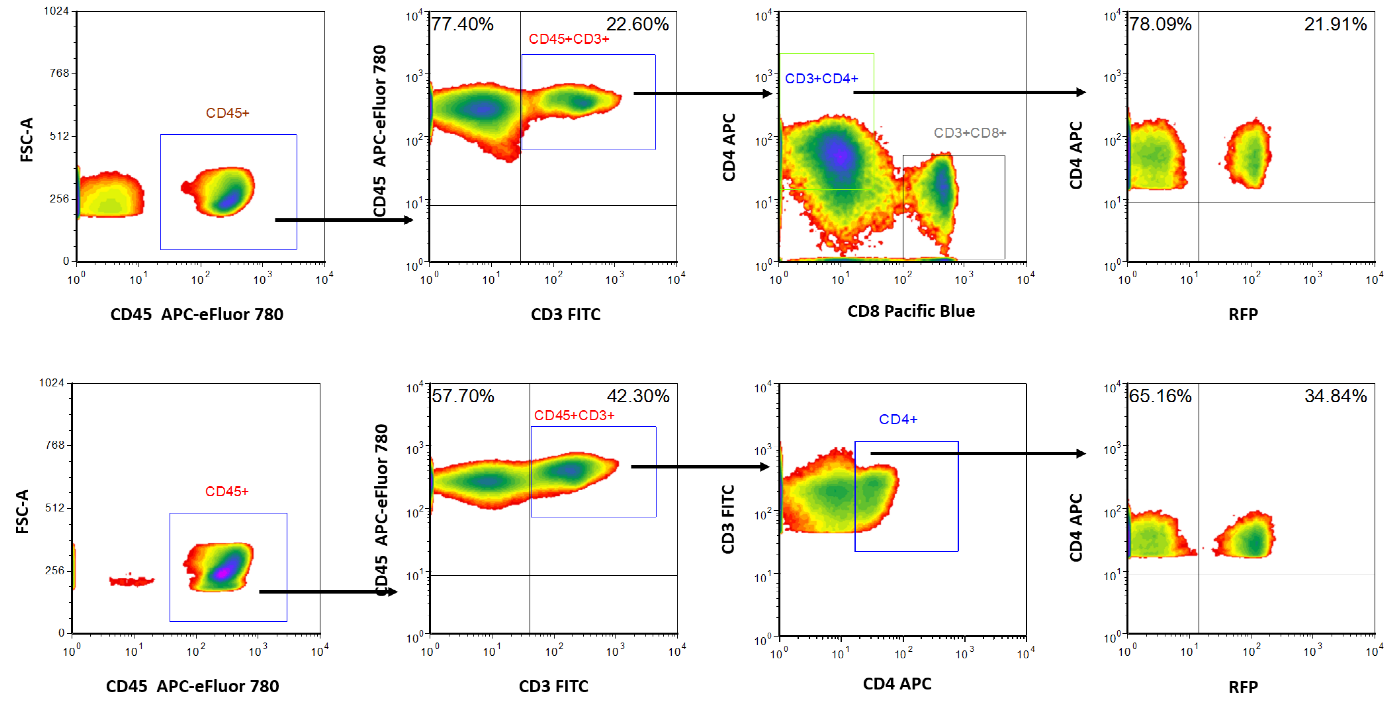
**

**Supplementary Figure 6: Gating strategies used in** *Foxp3^cre^ROSA^RFP^* mice. Figure exemplifying gating strategy to determine T_regs_ within **A)** lung and **B)** colon.

**
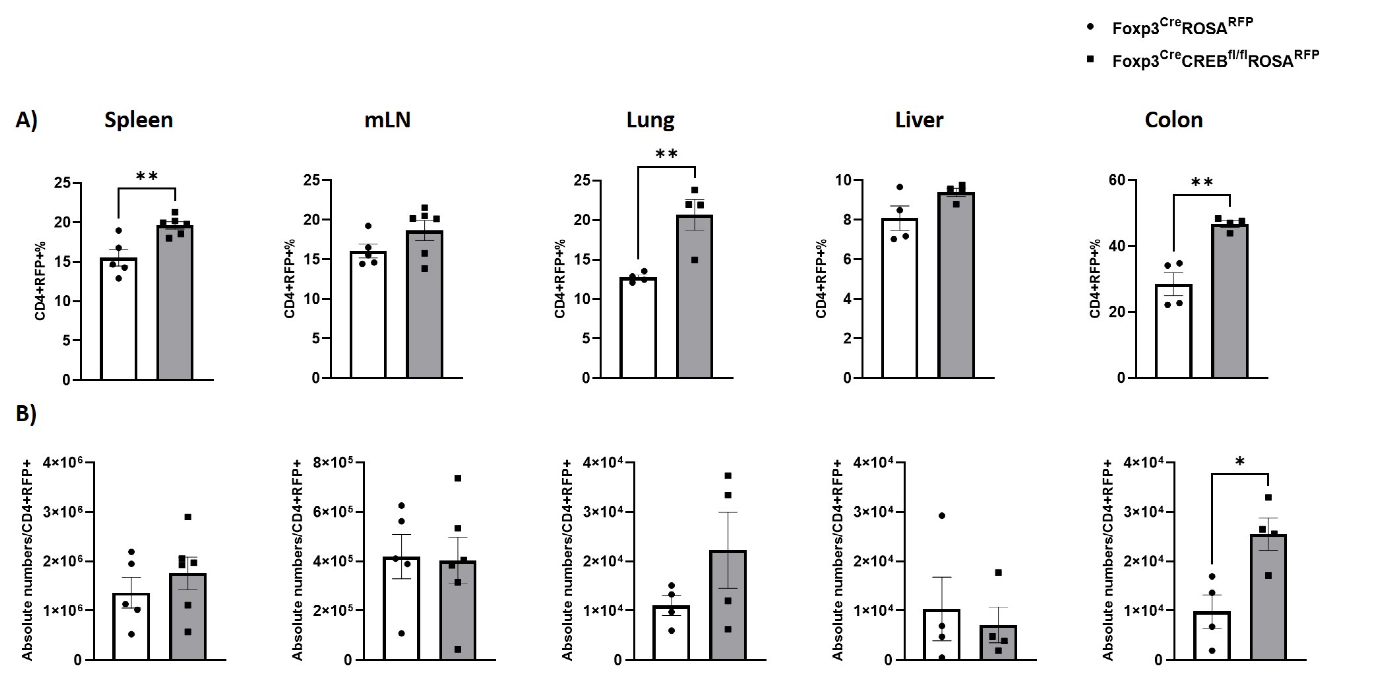
**

**Supplementary Figure 7:** Cell frequencies **A)** and absolute cell numbers **B)** of RFP+ cells in spleens, mLN (mesenteric lymph nodes), lungs, livers and colons of Foxp*3^cre^ROSA^RFP^* and *Foxp3^cre^CREB^fl/fl^ROSA^RFP^* mice. Unpaired Student t-test was performed. **p<0.01 and results are expressed as the mean ± SEM.

**Supplementary Figure 8:** Expression of CREB on T_eff_ and T_reg_ cell populations in Spleens, peripheral Lymph nodes, Lungs, Livers and colons of Foxp*3^cre^ROSA^RFP^* mice. ONE-way ANOVA test was performed. *p<0.05, **p<0.01, ***p<0.001, ****p<0.0001 and results are expressed as the mean ± SEM.


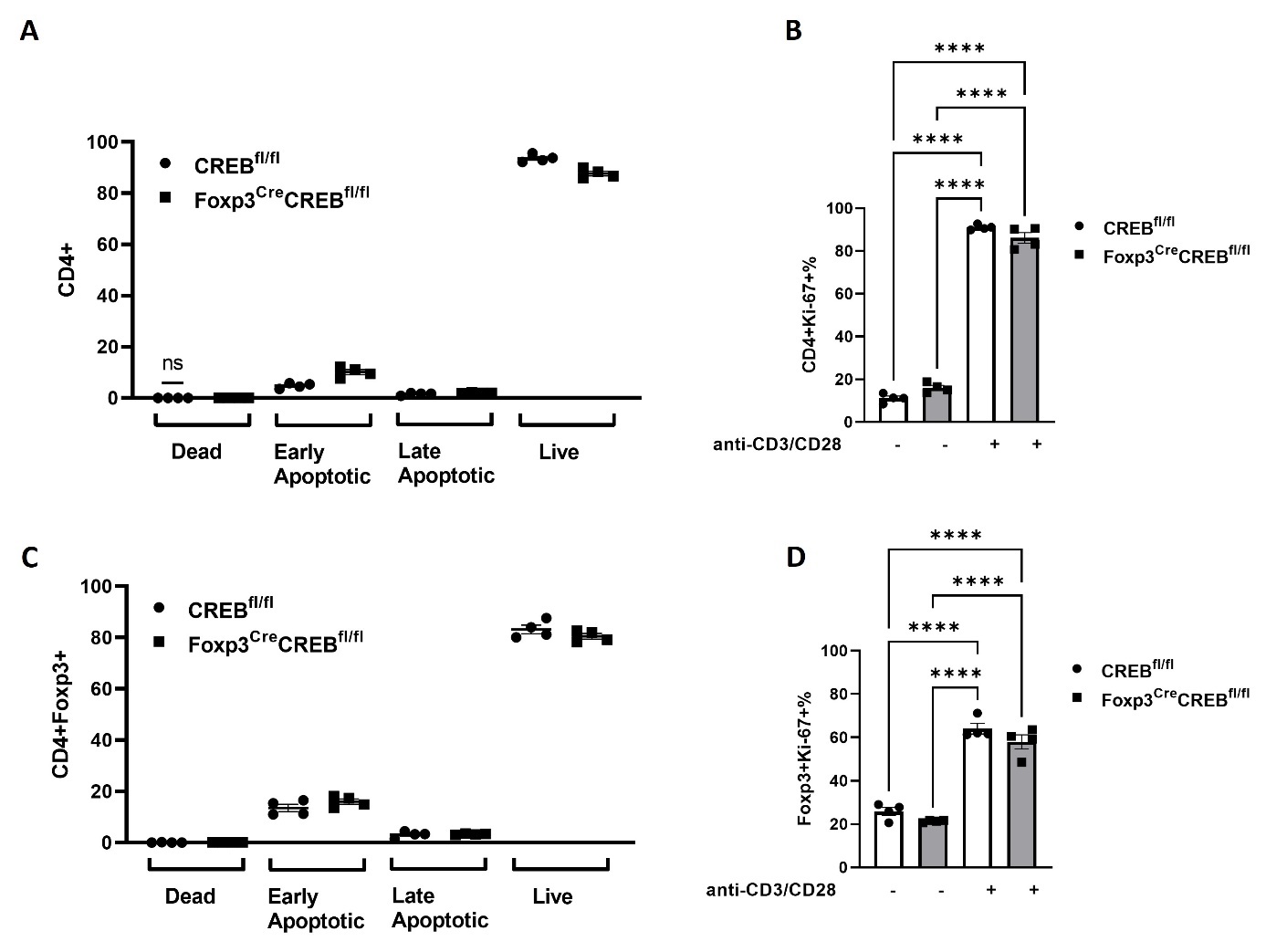


**Supplementary Figure 9:** Splenocytes of *WT and* *Foxp3^cre^CREB^fl/fl^* mice were stimulated with anti-CD3/CD28 for 2 days and stained for cell survival and proliferation. Annexin and Fixable viability-stained cells of **A)** CD4+ & **C)** CD4+Foxp3+ cells. Ki-67-stained cell percentages of **B)** CD4+ & **D)** CD4+Foxp3+ cells. ONE way-ANOVA test was performed. ns – p>0.05, ****p<0.0001 and results are expressed as the mean ± SEM.


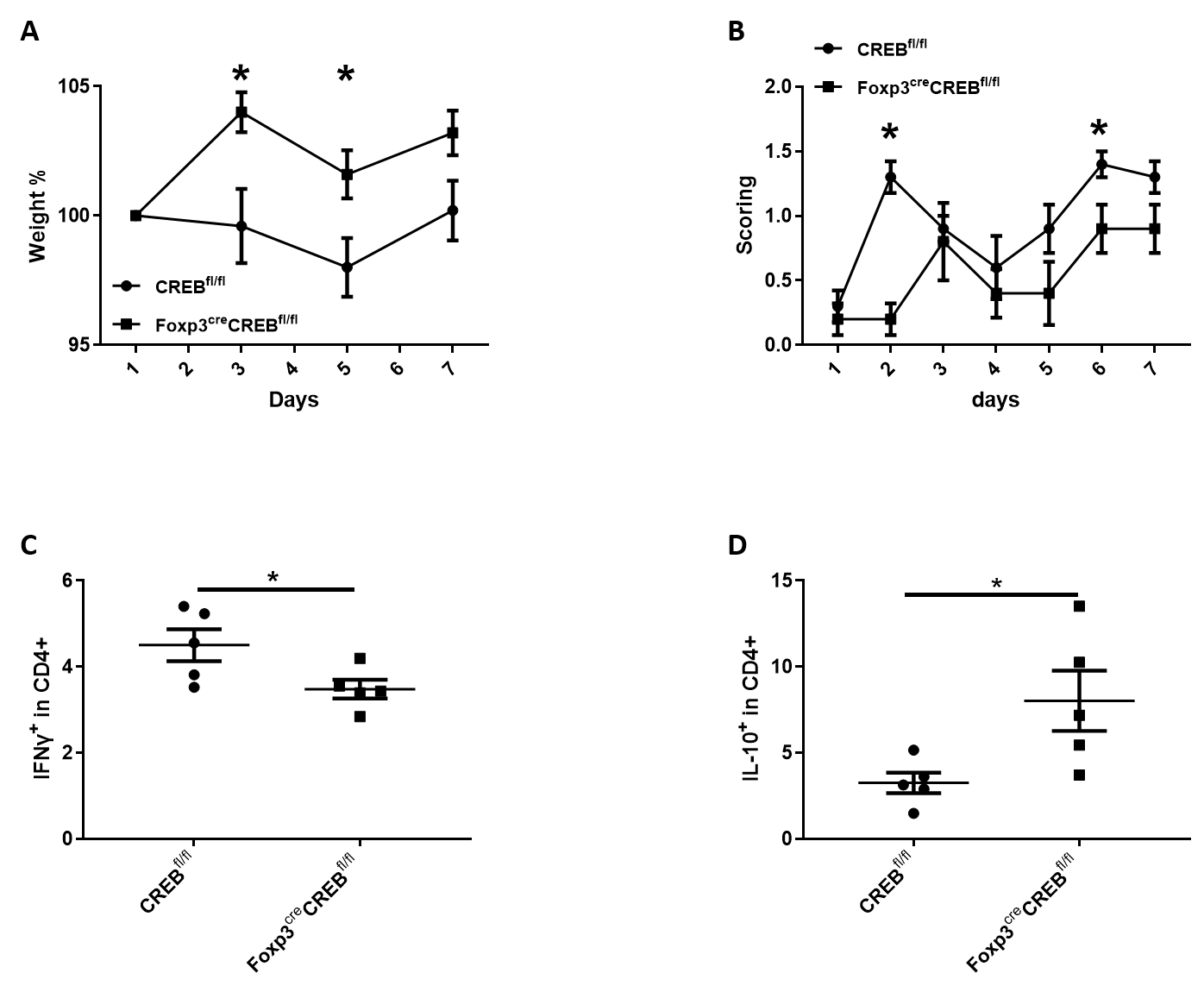


**Supplementary Figure 10:** ***Foxp3^cre^CREB^fl/fl^* mice only develop moderate signs of inflammation in an EAE model.** EAE was induced in female mice (*N = 5* CREB^fl/fl^ and *N = 5* *Foxp3^cre^CREB^fl/fl^* mice). A) Loss of weight and B) EAE score were measured beginning after onset of symptoms. C) Statistical analysis of IFN-γ^+^ cells within splenic CD4^+^ T cells. D) Statistical analysis of IL-10^+^ cells within splenic CD4^+^ T cells, two tailed unpaired t-test, each dot represents one animal, error bars SEM, *N = 5* CREB^fl/fl^ and *N = 5* *Foxp3^cre^CREB^fl/fl^*.


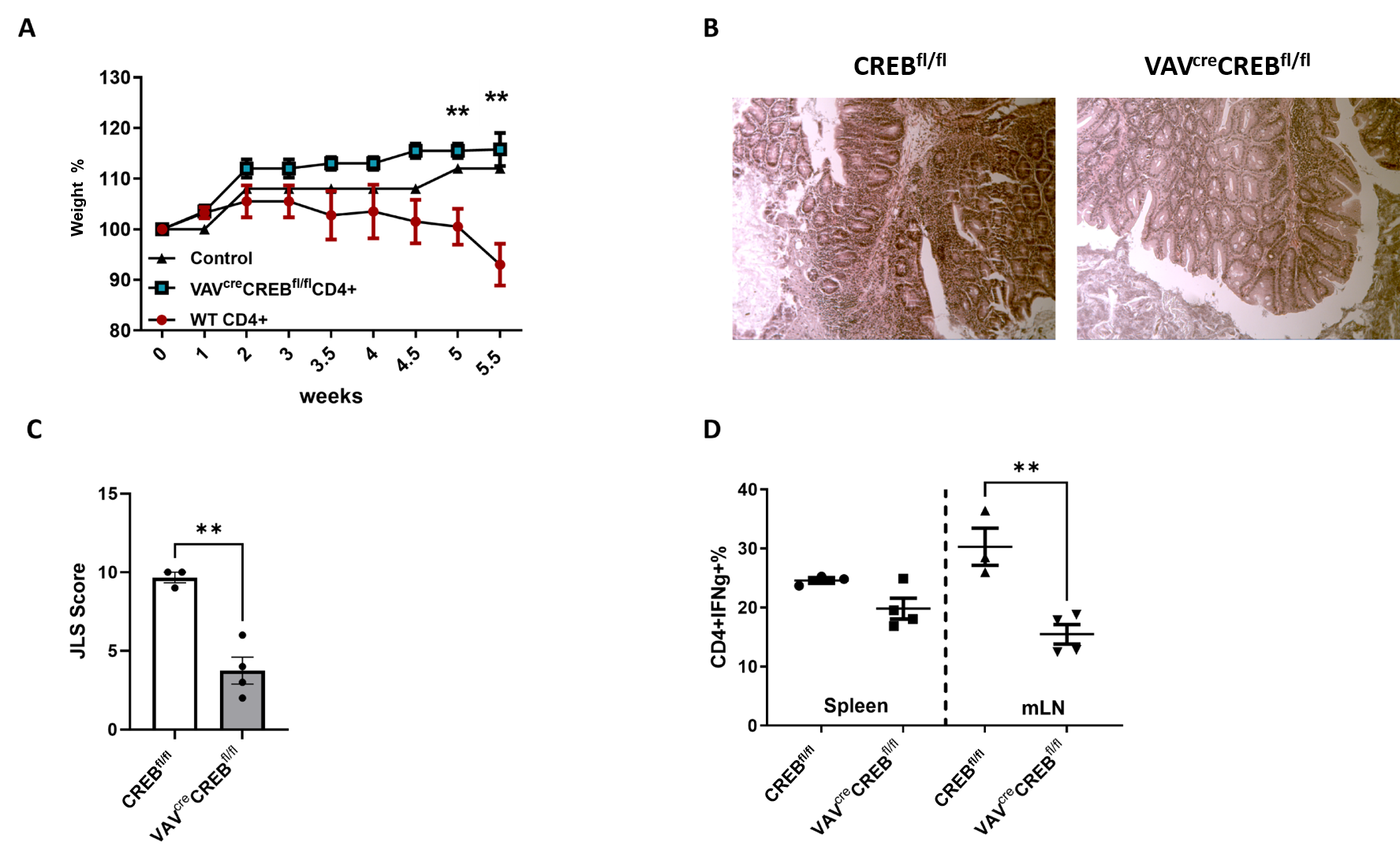


**Supplementary Figure 11: *Vav^cre^Creb^fl/fl^* CD4+ T cells induce only moderate inflammation in experimental colitis.** Rag^-/-^ mice were adoptively transferred with CD4^+^ wild-type cells (*CREB^fl/fl^* CD4^+^) or *Vav^CRE^CREB^fl/fl^* CD4^+^ T cells. Untreated Rag^-/-^ mice were used as controls (K). Mice were weighed and sacrificed 5 weeks after transfer. (A) Body weight as a percent of starting weight. (B) Representative photomicrographs of hematoxylin and eosin (H&E)-stained colon sections imaged using a 10x objective. C) Results of histological TJL (The Jackson Laboratory Score) scoring of colon sections (one independently performed experiments, *CREB^fl/fl^* recipients N = 3, *VAV^cre^CREB^fl/fl^* recipients N = 4). D) Statistical analysis of IFNy within CD4^+^ cells in spleen and mLNs (*CREB^fl/fl^* recipients N = 3, *VAV3^cre^CREB^fl/fl^* recipients N = 4), a two-tailed, unpaired t-test was used.
